# Natural history of *SLC11 *genes in vertebrates: tales from the fish world

**DOI:** 10.1186/1471-2148-11-106

**Published:** 2011-04-18

**Authors:** João V Neves, Jonathan M Wilson, Heiner Kuhl, Richard Reinhardt, L Filipe C Castro, Pedro NS Rodrigues

**Affiliations:** 1 Iron and Innate Immunity, Instituto de Biologia Molecular e Celular (IBMC), Rua do Campo Alegre 823, 4150-180 Porto, Portugal; 2 Centro Interdisciplinar de Investigação Marinha e Ambiental (CIIMAR), Rua dos Bragas 289, 4050-123 Porto, Portugal; 3 Max-Planck-Institute for Molecular Genetics, Ihnestraße 63-73, 14195 Berlin, Germany; 4 Instituto de Ciências Biomédicas Abel Salazar (ICBAS), Universidade do Porto, Largo Prof. Abel Salazar 2, 4099-003 Porto, Portugal

## Abstract

**Background:**

The *SLC11A1/Nramp1 *and *SLC11A2/Nramp2 *genes belong to the *SLC11/Nramp *family of transmembrane divalent metal transporters, with *SLC11A1 *being associated with resistance to pathogens and *SLC11A2 *involved in intestinal iron uptake and transferrin-bound iron transport. Both members of the *SLC11 *gene family have been clearly identified in tetrapods; however *SLC11A1 *has never been documented in teleost fish and is believed to have been lost in this lineage during early vertebrate evolution. In the present work we characterized the *SLC11 *genes in teleosts and evaluated if the roles attributed to mammalian *SLC11 *genes are assured by other fish specific *SLC11 *gene members.

**Results:**

Two different *SLC11 *genes were isolated in the European sea bass (*Dicentrarchus. labrax*), and named *slc11a2-α *and *slc11a2-β*, since both were found to be evolutionary closer to tetrapods *SLC11A2*, through phylogenetic analysis and comparative genomics. Induction of *slc11a2-α *and *slc11a2-β *in sea bass, upon iron modulation or exposure to *Photobacterium damselae *spp. *piscicida*, was evaluated in *in vivo *or *in vitro *experimental models. Overall, *slc11a2-α *was found to respond only to iron deficiency in the intestine, whereas *slc11a2-β *was found to respond to iron overload and bacterial infection in several tissues and also in the leukocytes.

**Conclusions:**

Our data suggests that despite the absence of *slc11a1*, its functions have been undertaken by one of the *slc11a2 *duplicated paralogs in teleost fish in a case of synfunctionalization, being involved in both iron metabolism and response to bacterial infection. This study provides, to our knowledge, the first example of this type of sub-functionalization in iron metabolism genes, illustrating how conserving the various functions of the SLC11 gene family is of crucial evolutionary importance.

## Background

The solute carrier family 11 (*SLC11*) is a gene family of divalent metal transporters, composed by two functional paralogs, *SLC11A1 *and *SLC11A2*. The first member of the *SLC11 *family, SLC11A1, also known as the natural resistance-associated macrophage protein 1 (NRAMP1), is a divalent cation/proton transporter, which has been proposed to function as either a symporter [1,2] or an antiporter [3,4]. Its expression is almost exclusively restricted to the membrane of late endosomes and lysosomes of immune cells of myeloid lineages (neutrophils, macrophages, dendritic cells) [5,6] and to neuronal cells [7]. SLC11A1 was first found to play a crucial role in the defense against several unrelated pathogens in mice, such as *Mycobacteria, Leishmania *and *Salmonella *[8-10], and several studies have shown that polymorphisms in SLC11A1 are involved in many infectious [11-15] and autoimmune [16-20] diseases in humans. However, the resistance mechanisms attributed to SLC11A1 are still not fully understood [21,22].

The second member of the *SLC11 *family, SLC11A2, is also referred to as natural resistance-associated macrophage protein 2 (NRAMP2), divalent cation transporter 1 (DCT1) or divalent metal transporter 1 (DMT1). SLC11A2 is a divalent cation/proton symporter [23], with a ubiquitous expression [24-26]. It is known to take up iron from the intestinal brush border in mammals and has been linked to transferrin-dependent iron transport from acidified endosomes to the cytosol in many different tissues [1,23,25]. Polymorphisms in SLC11A2 are known to underline microcytic anemia in mice and rats, resulting from an impairment of iron recycling and intestinal absorption [27,28].

SLC11 homologs have been found in many distant evolutionarily related groups, such as humans [29,30], mice [9,24], rats [23], birds [31], fishes [32-36], insects [37], nematodes [38], plants [39], yeast [40] and even bacteria [41]. Complete *slc11 *mRNA coding sequences for teleost fishes have been published in the last few years. Single genes were described in carp (*Cyprinus carpio*) [42], channel catfish (*Ictalarus punctatus*) [43], zebrafish (*Danio rerio*) [44], striped bass (*Morone saxatilis*) [32], Japanese flounder (*Paralichthys olivaceus*) [33], turbot (*Scophthalmus maximus*) [35] and red sea bream (*Pagrus major*) [34], while two copies have been described in rainbow trout (*Oncorhynchus mykiss*) [45] and fugu (*Takifugu rubripes*) [36], with evidence from other teleosts available in various genome databases.

Most animal studies, particularly in teleost fishes, are focused on gene isolation and constitutive expression analysis, with little information on evolutionary and functional aspects. Furthermore, a complex picture emerges from the comparison of phylogenetic and expression studies between fishes and mammals [36]. In fact, little is known about the structure and function of SLC11 in lower vertebrates, although some studies provide evidence for a role of teleost Slc11a2 orthologs in the nutritive metal uptake in the intestine [46,47], and also an involvement in the response to bacterial infection. It has been shown that *slc11 *mRNA levels are elevated in response to lipopolysaccharide (LPS) and *Edwardsiella ictaluri *[43,48] in channel catfish, to *Vibrio angillarum *in turbot [35] and red sea bream [34] and to *Mycobacterium *in striped bass [32]. However no research has yet elucidated this complex pattern of phylogenetic relationships and functional roles of teleost and mammalian *SLC11 *genes and no explanations are provided as to why it seems that a homolog of the mammalian SLC11A2 is performing the functions attributed to SLC11A1.

A complement of two *SLC11 *genes is shared between mammals and teleosts. Whilst it is known that mammalian *SLC11A1 *and *SLC11A2 *have likely resulted from genome duplications in early vertebrate ancestry (2R) [36,49,50], the potential role of the teleost fish-specific genome duplication (3R) [51,52] in the evolutionary history of this gene family has not been considered. A comprehensive synteny study could thus help to improve our understanding of the evolution and functional specialization of these genes in teleost fish.

European sea bass (*Dicentrarchus labrax*) was selected as the teleost model for this study due to the growing amount of data on its immune system [53], the possibility of making use of its partially sequenced genome [54,55] and our previous experience with sea bass models of infection and iron modulation [56-59]. Sea bass is also an important marine aquaculture species in Europe, afflicted by several diseases such as pasteurellosis and vibriosis. Since the early 1980s, its production has risen considerably [60] evolving from extensive culture units to semi-intensive or intensive systems. This massive fish concentration leads to an increase in organismal stress and as a consequence fish defenses get compromised, making them more susceptible to pathogen attack. Isolation and characterization of the *slc11 *gene(s) in sea bass, as a candidate gene(s) for host defense to infection with pathogens, may be of great benefit to better understand its role in the immune system and to the selection of disease resistant stocks [61]. Moreover, sea bass is part of the Acanthopterygii superorder, which includes stickleback, tetraodon and fugu, organisms that have their genome fully sequenced, making possible a number of comparative genetic studies.

The aims of this study were to identify and characterize the sea bass *SLC11 *homologs, clarify their evolutionary history and to determine their functional roles, in particular those related with the host iron metabolism and resistance to infection. We evaluated the modulation of *SLC11 *gene(s) expression in sea bass upon iron modulation (iron deficiency and overload) or exposure to *Photobacterium damselae *spp. *piscicida*, in *in vivo *or *in vitro *experimental models. We expect that this approach should provide an insight on the evolutionary history of the *SLC11 *genes in the vertebrata subphylum.

## Results

### Southern Blot

**Figure 1:**
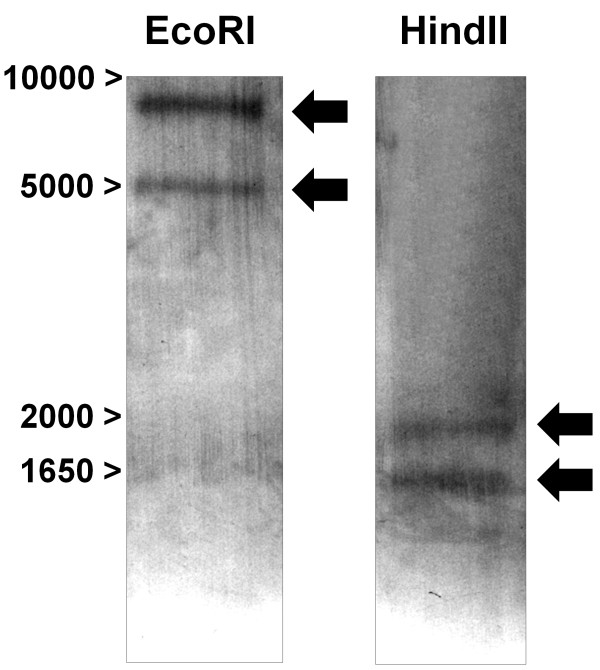
**Southern blot analysis**. 10 μg of genomic DNA were independently digested with EcoRI or HindII and hybridized with a DIG-labelled *slc11 *RNA probe. Molecular weights (bp) are indicated in the left margin.

In order to determine the number of copies of *slc11 *genes in the sea bass genome, a southern blot analysis was performed (Figure 1). After independent digestion of 10 μg of genomic DNA with EcoRI or HindII and hybridization with a *slc11 *DIG-labeled probe, different hybridization bands were visible. No uncut products were observed. Whether digested with EcoRI or HindII, two different hybridization bands were visible, suggesting the existence of two copies of the *slc11 *genes in the sea bass genome.

### Sea bass *slc11 *transcripts

Five different sea bass *slc11 *transcripts were obtained by primer walking and 5'/3' RACE with liver, spleen and intestine cDNA and analysis of whole-genome shotgun contigs (Max Planck Institute for Molecular Genetics).

**Figure 2:**
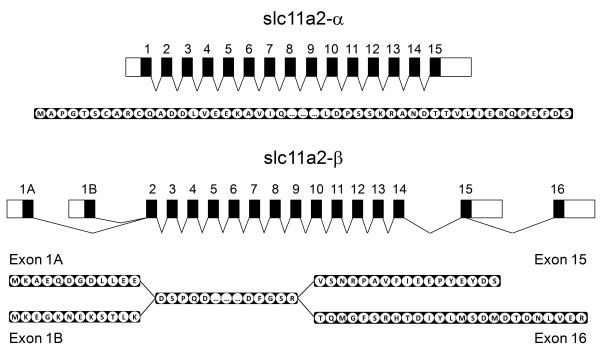
**Schematic representation of *slc11a2-α *and *slc11a2-β *transcripts and putative proteins**. *Slc11a2-α *produces a single transcript, encoding a 560 aa protein. *Slc11a2-β *produces 4 transcripts, encoding 4 putative proteins, 2 of 554 aa (*β1 *and *β2*, from exons 1A or 1B to exon 15, respectively) and 2 of 562 aa (*β3 *and *β4*, from exons 1A or 1B to exon 16, respectively). Difference in size results from an alternative splice site in exon 15 and replacement of its final 17 aa for 25 aa encoded by exon 16. Exons are represented as black boxes, UTRs as white boxes.

One transcript was named *slc11a2-α *and the other four transcripts were named *slc11a2-β1 *to *β4*, since they were found to share 1557 bp of their coding region (out of a total of 1665 bp for *β1/β2 *or 1689 bp for *β3/β4*), with the differences between them limited to the 5' and 3' endings (Table 1, Figure 2 and Additional File 1, Figure S1).

**Table 1:** Sea bass *slc11 *transcripts

Transcript	5' UTR (bp)	First Exon	Coding (bp)	Last Exon	3' UTR (bp)	Full lenght (bp)
*slc11a2-α*	116	1	1683	15	120	1919

*slc11a2-β1*	203	1A	1665	15	569	2437
*slc11a2-β2*	121	1B	1665	15	569	2355
*slc11a2-β3*	203	1A	1689	16	319	2211
*slc11a2-β4*	121	1B	1689	16	319	2129

Sizes of the untranslated regions and coding regions, as well as starting and ending exons for each transcript.

*Slc11a2-β1 *and *slc11a2-β2 *transcripts are of the same length regarding the coding region, although they differ in the first 34 bp, since they result from the alternative usage of either exon 1A (β1) or exon 1B (β2), both with 34 bp. They also have in common the final exon (exon 15), and the same 3' UTR (Table 1, Figure 2 Additional File 1, Figure S1). *Slc11a2-β3 *and *slc11a2-β4 *transcripts are also of the same length and also differ in the first 34 bp, due to alternative usage of either exon 1A (β3) or exon 1B (β4). Both are slightly larger than *slc11a2-β1 *and *slc11a2-β2*, since exon 15 presents an alternative splice site, losing the final 54 bp (out of 108 bp) and being partially substituted by the 78 bp of exon 16 (Table 1, Figure 2 and Additional File 1, Figure S1). *Slc11a2-β3 *and *slc11a2-β4 *also share the same 3' UTR, albeit different and smaller than the 3' UTR for *slc11a2-β1 *and *slc11a2-β2 *(Table 1, Figure 2 and Additional File 1, Figure S1).

### Genomic Organization

The genomic organization of sea bass *slc11a2-α *and *slc11a2-β *was analyzed and compared with those of other fishes, amphibians and mammals (see Additional File 2, Figure S2). Exon/intron boundaries were determined by comparison of cDNA, genomic DNA and putative amino acid sequences, splice-site consensus matching and analysis of whole-genome shotgun contigs (Max Planck Institute for Molecular Genetics).

Comparison of genomic DNA sequences with the previously obtained cDNA sequences showed that *slc11a2-α *consists of 15 exons and 14 introns, with a single initiator methionine and stop codon. The genomic interval from the initiator methionine to the stop codon is 7842 bp. Comparison of genomic DNA sequences with the cDNA sequences of the four *slc11a2-β *transcripts showed that those four transcripts result not from four different *slc11a2-β *genes, but rather from the alternative splicing of two 5' exons and two 3' exons of a single *slc11a2-β *gene (as already suggested by the southern blot results), similar to what happens with human *SLC11A2 *[62,63]. The *slc11a2-β *gene comprises a total of 17 exons, with two initiator methionines and two stop codons. Differences in the 5'-termini are generated by alternative promoters with subsequent, mutually exclusive splicing of the respective first exons to exon 2, whereas differences in the 3'-termini are due to alternative splicing of exon 15, which presents an alternative 5' donor site (corroborated using Alternative Splice Site Predictor [64,65]). In two isoforms, this leads to the reading of exon 15 and in the other two isoforms to the partial reading of exon 15 and also exon 16 (Figure 2). The genomic interval from the first initiator methionine in exon 1A to the second stop codon in exon 16 is 10543 bp.

For both α and β genes, exons 2-14 present a high homology with the equivalent exons from other fish and mammals, whereas exons in the N- and C-terminus are variable in size and sequence. Intron sizes, much like in fugu and tetraodon, are reduced when compared with mammalian homologs. Sea bass *slc11a2-α *and *slc11a2-β *present a compaction factor of 1.8× and 1.4× to human *SLC11A1 *and 4.6× and 3.4× to human *SLC11A2*, respectively.

No clear sequences matching the iron responsive element (IRE) motifs, commonly found in mammalian *SLC11A2*, were identified in any of the 3' regions of either *slc11a2-α *or *slc11a2-β*. However, one IRE motif was found in the 5' region of *slc11a2-β *(5'-UGUCUGUGUCAGAGAACAUGGUG-3'), with an upstream distance to the start codon of 508 bp, using RegRNA [66,67] and corroborated manually.

Full-length genomic sequences of sea bass *slc11a2-α *and *slc11a2-β *were deposited in GenBank with accession numbers HQ451945 (*slc11a2-α*) and HQ451946 (*slc11a2-β*).

### Structure analysis of sea bass Slc11 putative proteins

To predict the functionality of sea bass Slc11 putative proteins, we compared them with other known SLC11 proteins, and analyzed them using several bioinformatics tools (listed at ExPASy [68]), searching for characteristic features of this protein family.

The complete open reading frame of sea bass *slc11a2-α *comprises a single transcript of 1683 nucleotides, encoding a putative protein of 560 residues (Figure 2 and Additional File 1, Figure S1), with a predicted molecular mass of 61.6 kDa. The complete open reading frame of sea bass *slc11a2-β *comprises 4 different transcripts, encoding 4 putative proteins (Figure 2 and Additional File 1, Figure S1) that result from the alternative usage of two 5' exons encoding distinct N-termini and the alternative splicing of two 3' exons encoding distinct C-termini of the proteins. Two transcripts of 1665 nucleotides, named *slc11a2-β1 *(from exon 1A to exon 15) and *slc11a2-β2 *(from exon 1B to exon 15), and two transcripts of 1689 nucleotides, named *slc11a2-β3 *(from exon 1A to exon 16) and *slc11a2-β4 *(from exon 1B to exon 16), encode putative proteins of 554 and 562 residues, with predicted molecular masses of 61.2 and 62.2 kDa, respectively.

The 8 amino acid discrepancy in length results not from the substitution of exon 15 for exon 16, but rather from an alternative splice site in exon 15. Only the final 17 amino acids encoded by exon 15 (out of 35) are replaced by the 25 amino acids encoded by exon 16 (figure 2). Both exon 1A and exon 1B have the same size and encode the same number of amino acids.

**Figure 3:**
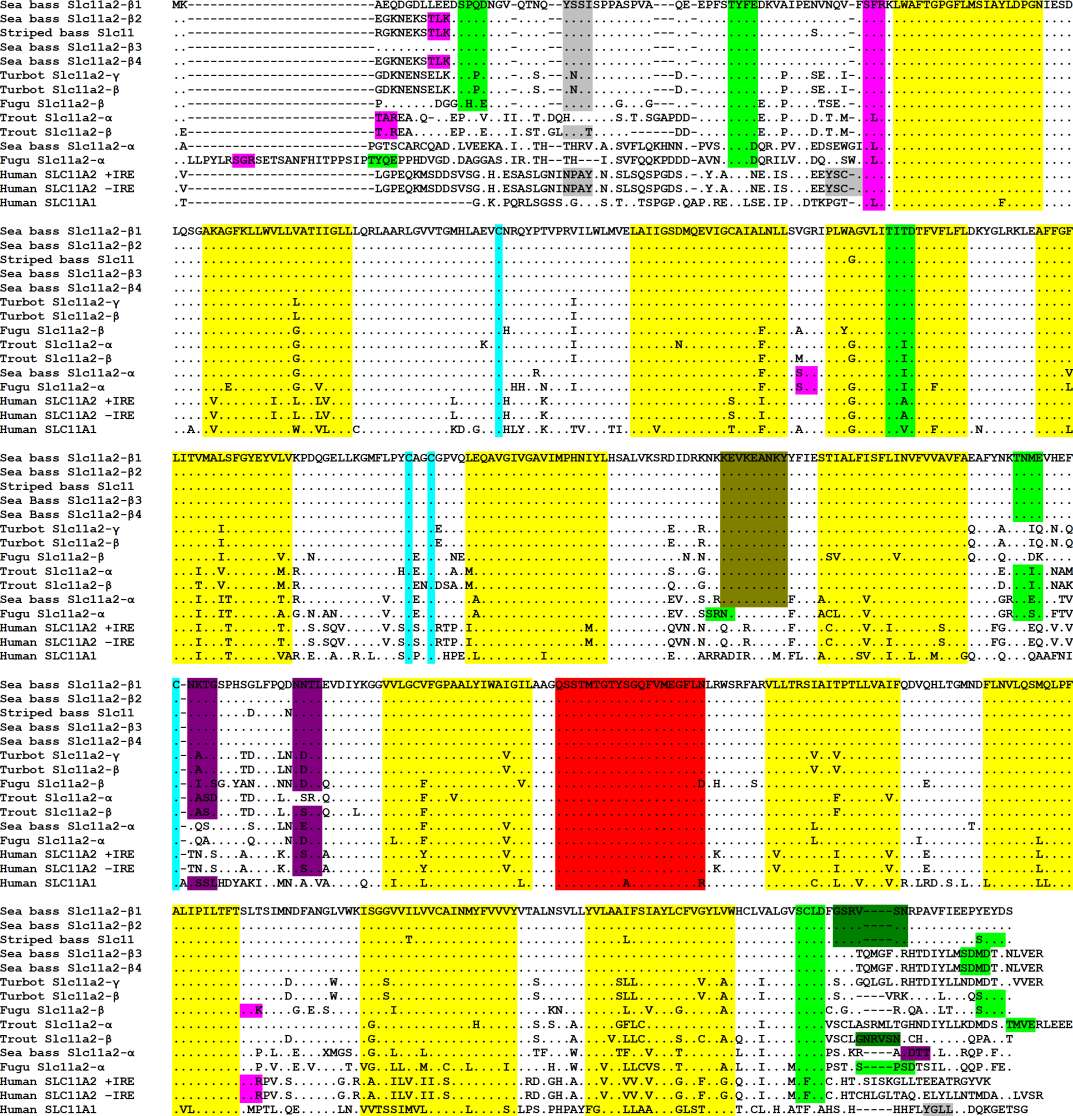
**Amino acid alignment**. Sea bass Slc11a2-α and Slc11a2-β's were aligned with striped bass Slc11 (AAG31225), turbot Slc11a2-β (ABB73023) and Slc11a2-γ (ABE97051), fugu Slc11a2-α (CAD43050) and Slc11a2-β (CAD43051), trout Slc11a2-α (AAD20721) and Slc11a2-β (AAD20722), human SLC11A1 (NP_000569), human SLC11A2 +IRE (NP_000608) and human SLC11A2 -IRE (AAC21459). Identical residues and gaps are indicated by dots and dashes, respectively. Signature features and putative motifs and highlighted as follows: yellow, transmembrane domains; red, conserved transport motif; violet, N-linked glycosilation site; pink, protein kinase C phosphorylation site; light green, casein kinase II phosphorylation site; olive green, tyrosine kinase phosphorylation site; gray, tyrosine based sorting signal; cyan, conserved cysteine residues; dark green, N-myristoylation sites

**Figure 4:**
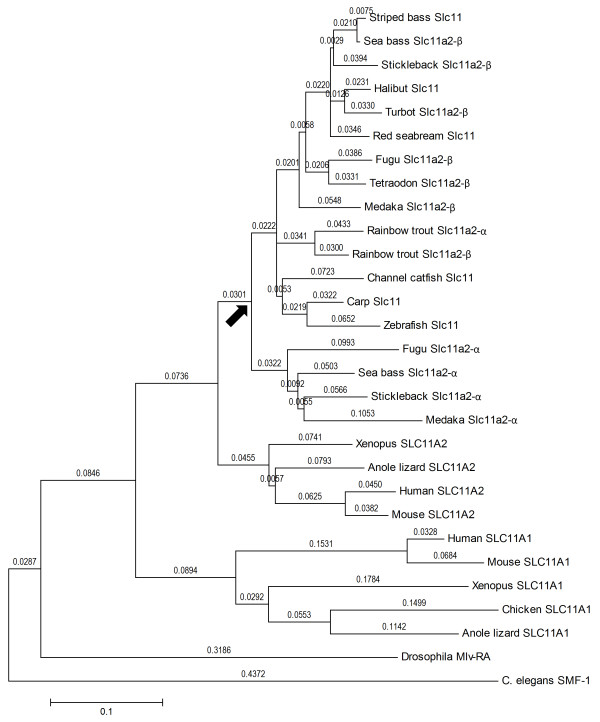
**Molecular Phylogenetic analysis by Maximum Likelihood method**. The evolutionary history was inferred by using the Maximum Likelihood method based on the JTT matrix-based model. The bootstrap consensus tree inferred from 1000 replicates is taken to represent the evolutionary history of the taxa analyzed. Initial tree(s) for the heuristic search were obtained automatically as follows. When the number of common sites was < 100 or less than one fourth of the total number of sites, the maximum parsimony method was used; otherwise BIONJ method with MCL distance matrix was used. The tree is drawn to scale, with branch lengths measured in the number of substitutions per site (above the branches). The analysis involved 29 amino acid sequences. All positions containing gaps and missing data were eliminated. There were a total of 408 positions in the final dataset. Arrow indicates the duplication point of α and β isoforms in teleosts.

Comparison with other teleost and mammalian species SLC11 proteins showed that the signature features of the SLC11 family [36,42,45,69] can also be found in the sea bass (Figure 3 and Additional File 1, Figure S1): twelve putative transmembrane domains (TMD), a conserved transport motif (between TMD8 and TMD9), cysteine residues in loops 2, 5 and 7. Other motifs observed in previously described SLC11 proteins, including some exclusive to teleost fish, are also present in sea bass. Two N-linked glycosylation sites (N-X-S/T-X), located in loop 7, are present in all Slc11a2-β forms, but only one is present in Slc11a2-α loop 7, with another located in the C terminus. A conserved protein kinase C phosphorylation site (S/T-X-R/K) was also found immediately before TMD1, and one other was only found to be shared by sea bass Slc11a2-α and fugu Slc11a-α, in loop 3. A third protein kinase C phosphorylation site was found to be present in the 5' cytoplasmatic extremity of sea bass Slc11a2-β2 and Slc11a2-β4, striped bass Slc11, fugu Slc11a2-α and trout Slc11a2-α and β. Six, five and four casein kinase II phosphorylation sites (S/T-XX-D/E) were found in Slc11a2-β(3,4), Slc11a2-β(1,2) and Slc11a2-α, respectively. One tyrosine kinase phosphorylation site (R/K-XX(or XXX)-D/E-XX(or XXX)-Y) in loop 6 was found to be conserved in teleosts, with the exception of fugu Slc11a-α. One tyrosine based sorting signal (NPXY or YXXΦ; where Φ is a bulky hydrophobic residue) in the N terminus was also found to be conserved in teleosts, with the exception of the alpha forms of trout, sea bass and fugu. Several N-myristoylation sites **(**G-{EDRKHPFYW}-XX-[STAGCN]-{P}) were also found to be conserved among all Slc11a2-β forms (data not shown), but one site was only found to be present in the 3' cytoplasmatic extremity of sea bass Slc11a1-β(1,2), as well as striped bass Slc11 and Rainbow trout Slc11a2-β (figure 3). All phylogenetic analysis, performed with the maximum-likelihood, maximum-parsimony, neighbor-joining and Bayesian inference methods were found to be consensual, placing sea bass Slc11a2-β clustered with other fishes Slc11a2-β and Slc11 (Figure 4 and Additional File 3, Figure S3) and sea bass Slc11a2-α clustered with other fishes Slc11a2-α, with the exception of trout Slc11a2-α. As with all other teleost fish Slc11 homologs described so far, sea bass Slc11 homologs are also closer to mammalian SLC11A2 than to SLC11A1.

### Paralogy and synteny

**Figure 5:**
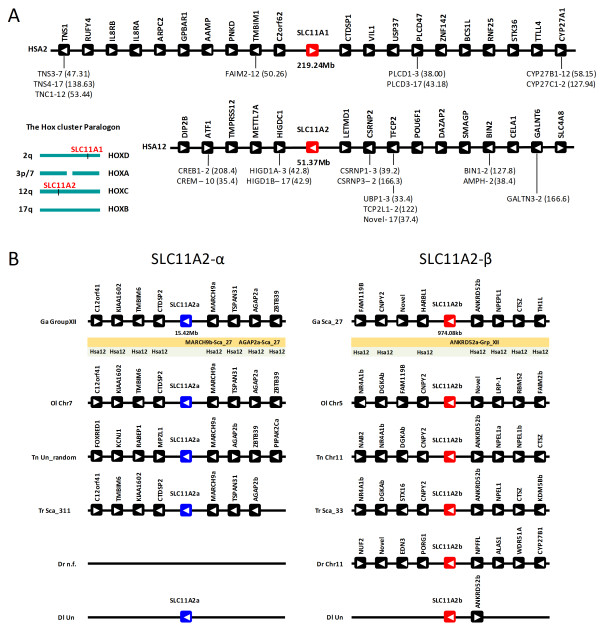
**Chromosomal location of the human *SLC11A1 *and *SLC11A2 *genes (A) and *SLC11 *gene *loci *in teleost species (B)**. (A) Paralogs of gene families with multiple members are shown below each ORF, with distance in Mb to the p telomere of the respective chromosome. (B) Below the *G. aculeatus loci*, the location of teleost specific 3R paralogs is shown; also the genomic mapping position of human orthologs is presented. Ga - *Gasterosteus aculeatus*, Dr - *Danio rerio*, Ol - *Oryzias latipes*, Tn - *Tetraodon nigroviridis*, Tr - *Takifugu rubribes*, Dl - *Dicentrarchus labrax*. Arrows denote gene orientation.

To determine the evolutionary history of the *SLC11 *gene family in vertebrates, we explored in detail the genomic "context" of both human *SLC11A1 *and *SLC11A2 *paralogs. Other authors [36] have previously suggested that *SLC11A1 *and *SLC11A2 *genes might have originated from the duplications of the genome in vertebrate ancestry, the so-called 2R, given their location to the Hox chromosomes in Hsa2 and Hsa12 (Figure 5 A). We find strong support for this hypothesis. Within a 1Mb window in the vicinity of the *SLC11A1 *and *SLC11A2 *genes, various gene families have paralog members mapping to expected Hox chromosome regions. That is the case of *TMBIM1*, which maps close to the *SLC11A1 *paralog in Hsa2, while *FAIM2 *maps to Hsa12. *TNS1 *maps to the left end of the window of the *SLC11A1 *gene, and has paralogs mapping to the three other human Hox genome locations. In the case of the *SLC11A2 *gene, 6 gene families show a consistent duplication pattern, with paralogs mapping to expected regions of Hox-linked paralogy, namely Hsa2q (Figure 5 A).

In the analysed teleosts a similar *SLC11 *gene complement is also found (with the exception of *D. rerio*). However, the precise orthology/paralogy relationships to the mammalian counterparts are yet to be firmly established. While the phylogeny clearly indicates that both fish genes strongly group with tetrapod *SLC11A2 *(Figure 4 Additional File 3, Figure S3), other features (e.g. subcellular localization) make the orthology assignment more contentious [36]. Syntenic data can be a powerful tool to facilitate the finding of orthologs and in the identification of duplication processes [70]. The abundance of genome data from teleosts allows us the use of mapping data to clarify the phylogenetic findings. We have analysed the genome locations and gene environment of the teleost *SLC11 *genes in zebrafish (*Danio rerio*), fugu (*Takifugu rubripes*), tetraodon (*Tetraodon nigroviridis*), medaka (*Oryzias latipes*) and stickleback (*Gasterosteus aculeatus*) (Figure 5 B). We find a strong degree of conservation between the gene arrangements of various fish species. The vicinity of the teleost *SLC11 loci *have their human ortholog equivalents mapping to the *SLC11A2 *chromosome, Hsa12q. Thus, we conclude that in agreement with the phylogenetic data, the genomic location of the teleost genes indicates that they are both *SLC11A2*-like. Furthermore, we observe that the mechanistic origin of the *SLC11 *genes is likely linked to the fish 3R genome duplication [71]. We find in the vicinity of both fish isoforms, gene families with duplicates mapping to both *SLC11 *fish chromosomal regions. For example, we noticed that *AGAP2 *gene family in medaka has one member mapping to chromosome 7 (along with one of the *SLC11 *isoforms), while a second isoform maps in chromosome 5 with the second isoform of teleost *SLC11*. The phylogenies of the genes families with duplicated members show that the duplication timing dates back to the origin of the teleosts (Additional File 4, Figure S4). Thus, we can safely conclude that the teleost genes are of the *SLC11A2 *type (which we name *slc11a2-α *and *slc11a2-β*), and resulted from the fish specific genome duplication.

### Constitutive expression of *slc11a2-α *and *slc11a2-β *in sea bass tissues

**Figure 6:**
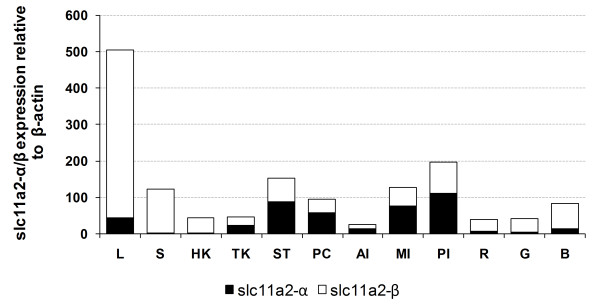
**Constitutive expression of *slc11a2-α *and *slc11a2-β***. Constitutive expression was measured in several sea bass organs by real-time PCR. Each sample was normalized to β-actin, calculated by the comparative C_T _method (2^-ΔΔC^T). L - liver; S -spleen; HK - head kidney; TK - trunk kidney; ST - stomach; PC - pyloric ceca; AI - anterior intestine; MI - mid intestine; PI - posterior intestine; R - rectum; G - gill; B - brain.

To gain some insight into sea bass *slc11 *basic functions, mRNAs constitutive expression of both forms was determined in relevant tissues. Constitutive expressions of *slc11a2-α *and *slc11a2-β *(all 4 isoforms) were evaluated by real-time PCR in several tissues, namely liver, spleen, head/trunk kidney, gill, brain, stomach, pyloric ceca, anterior/mid/posterior sections of the intestine and rectum (Figure 6). The liver was found to be the organ with the highest overall expression of the *slc11a2 *genes, followed by the mid and posterior portions of the intestine, stomach and spleen. Regarding the contribution of each *slc11a2 *form in the different tissues, there is a clear predominance of *slc11a2-β *in the liver, spleen, head kidney, rectum, gill and brain and a predominance of *slc11a2-α *in the stomach, pyloric ceca and all portions of the intestine.

### Constitutive expression of the four isoforms of *slc11a2-β *by semi-quantitative RT-PCR

**Figure 7:**
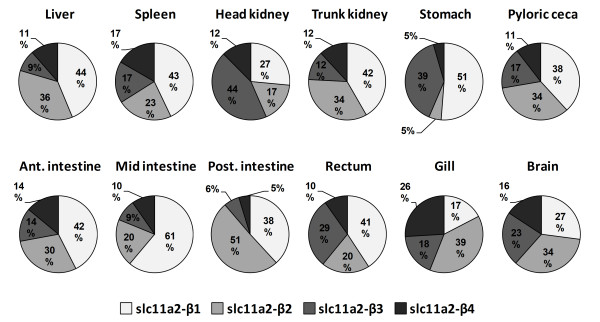
**Relative constitutive expression of the four *slc11a2-β *isoforms**. Constitutive expression of the *slc11a2-β *isoforms was determined in several sea bass organs, by optimized semi-quantitative PCR.

In order to assess the relative contribution of each β isoform to the overall *slc11a2-β *constitutive expression, a more thorough analysis was performed. Relative constitutive expressions of the four isoforms of sea bass *slc11a2-β *were determined by semi-quantitative PCR, since the distance between alternative 5' and 3' exons is too great to adequately use real-time PCR (Figure 7). The four isoforms were named *β1 *(from exon 1A to exon 15), *β2 *(from exon 1B to exon 15), *β3 *(from exon 1A to exon 16) and *β4 *(from exon 1B to exon 16).

In most of the tested tissues, there is a predominance of the *slc11a2-β1 *form, followed by *slc11a2-β2*. *Slc11a2-β3 *has a lower constitutive expression in most tissues, but presents significant expression in the head kidney, stomach and rectum, whereas *slc11a2-β4 *is the form with the lowest expression, although it represents 26% of the overall expression of *slc11a2-β *in the gill.

### *In situ *hybridization

An *in situ *hybridization was performed in several tissues to identify the subcellular distribution of sea bass *slc11 *mRNAs, to obtain further information useful in establishing parallelisms with their mammalian homologous counterparts, and to reiterate the results observed in the constitutive expression analysis.

**Figure 8:**
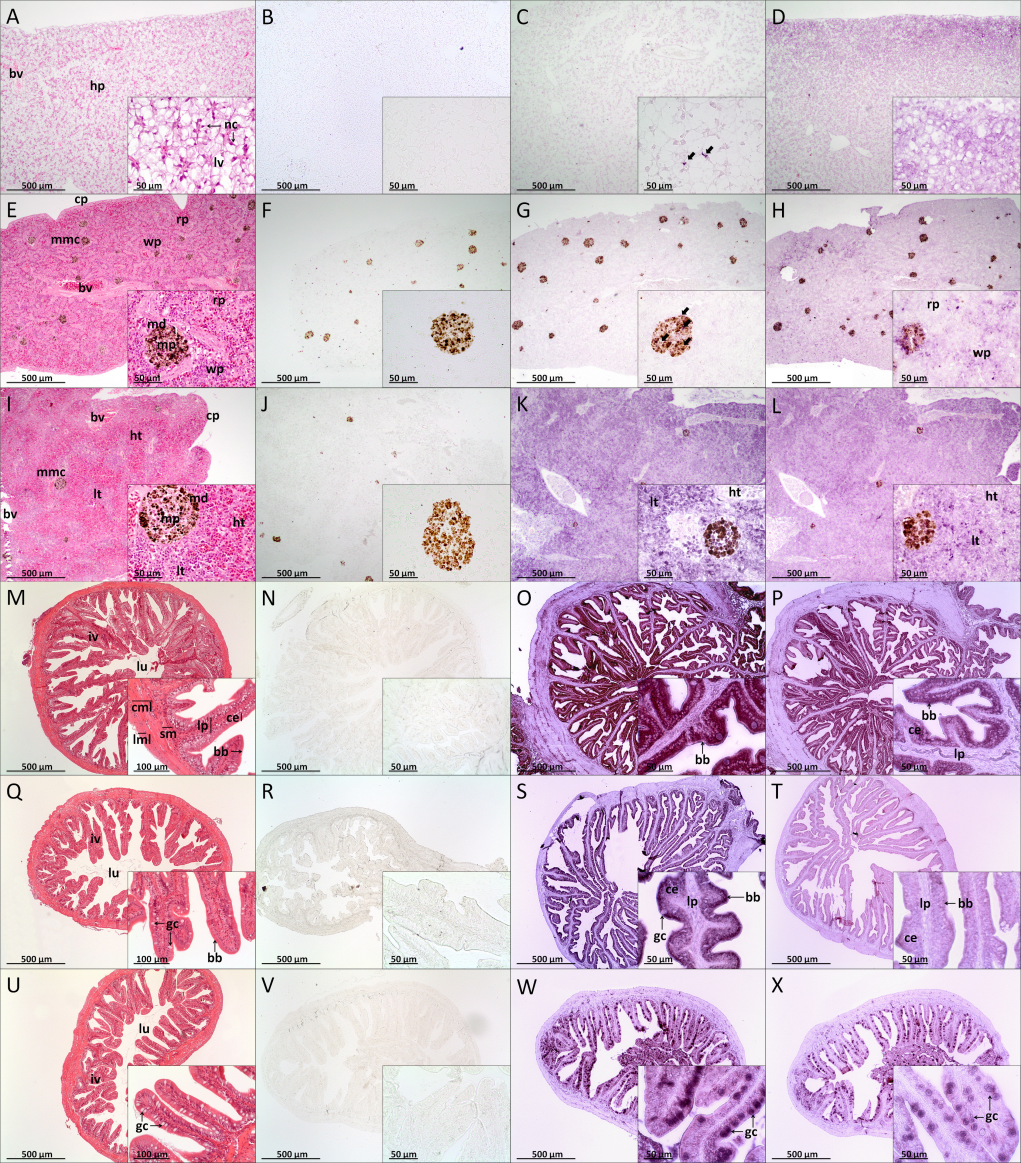
***In situ *hybridization**. A to D - liver; E to H - spleen; I to L - head kidney; M to P - anterior intestine; Q to T - mid intestine; U to X - posterior intestine. 1^st ^column - H&E staining; 2^nd ^column - hybridization with sense probes (control); 3^rd ^column - hybridization with *slc11a2-α *specific probe; 4^th ^column - hybridization with *slc11a2-β *specific probe. cp - capsule; rp - red pulp; wp - white pulp; mmc - melanomacrophage center; bv - blood vessel; md - melanin deposits; mc - macrophages; lt - lymphomyeloid tissue; ht - hematopoietic tissue; hp - hepatocytes; lv - lipidic vacuole: nc - nuclei; iv - intestinal villi; lu - lumen; lml - longitudinal muscle layer; cml - circular muscle layer; sm - submucosa; lp - lamina propria; ce - columnar epithelium; bb - brush border; gc - goblet cells. Thick arrows indicate points of *slc11a2-α *presence.

In the liver (Figure 8 A-D), *slc11a2-α *mRNA is very scarce, being detected in few hepatocytes whereas *slc11a2-β *in abundantly *slc11a2-α *mRNA is scarce, being only conspicuous in the melanomacrophage centers. *Slc11a2-β *on the other hand is abundant not only in the melanomacrophage centers but also in the spleen's white pulp. In the head kidney (Figure 8 I-L), both *slc11a2-α *and *slc11a2-β *have a similar distribution, being abundant in the melanomacrophage centers and the surrounding lymphomyeloid tissue, and to a much lesser degree in the hematopoietic tissue. In the intestine, *slc11a2-α *and *slc11a2-β *mRNA present similar patterns of distribution in the anterior (Figure 8 M-P) and mid section (Figure 8 Q-T). *Slc11a2-α *is found to be mostly concentrated in the apical (brush border) membrane of the enterocytes, whereas *slc11a2-β *presents a more homogeneous distribution, not only in enterocytes but also in other intestinal cells. However, in the posterior intestine (Figure 8 U-X), both forms of *slc11a2 *seem to be accumulated in or around the goblet cells, with limited presence in the enterocytes.

In all tissues, hybridization with sense probes (control) for either *slc11a2-α *or *slc11a2-β *produced no significant staining.

### Hematological parameters and tissue iron content in the *in vivo *experimental models

Several hematological and iron parameters were measured to validate the models of *in vivo *experimental iron modulation or infection with *Photobacterium damselae *spp. *piscicida*. During the experimental infection, mortality was monitored and found to be high, but with fish surviving past the final experimental time point, indicating a chronic infection (Additional File 5, Figure S5).

**Figure 9:**
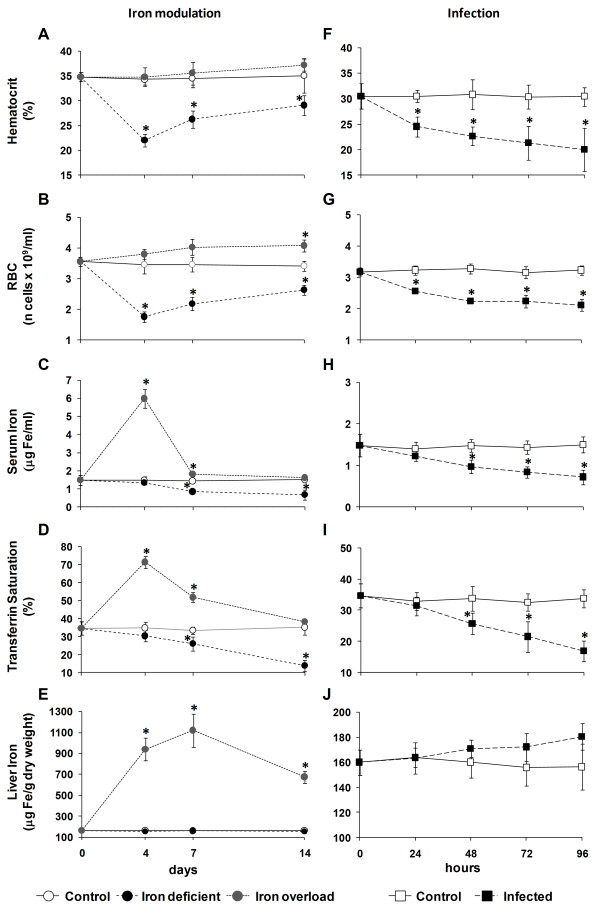
**Hematological parameters kinetics in iron modulated and infected fish**. *Iron modulation*: (A) hematocrit, (B) red blood cells count (RBC), (C) serum iron, (D) transferrin saturation and (E) liver iron; *Infection*: (F) hematocrit, (G) red blood cells count (RBC), (H) serum iron, (I) transferrin saturation and (J) liver iron. Values are expressed as means ± S.D. (n = 5 or n = 6 for iron modulation or infection, respectively). Samples were collected at 4, 7 and 14 days after iron modulation or 24, 48, 72 and 96 hours post-infection. Untreated fish were used as a 0-day control (n = 5). Differences from the controls were considered significant for *p < 0.05.

In the iron overloaded animals, no changes were observed in hematocrit (Figure 9 A), and a small but significant increase was observed in the RBC count during the course of the experiment (Figure 9 B). Increases in serum iron and transferrin saturation (Figure 9 C-D) were also observed, the highest levels at 4 days after overload, with decreases during the course of the experiment and recovery to control levels at 14 days. Liver iron levels were increased (Figure 9 E), with the maximum value at 7 days post-modulation and a decrease towards 14 days, but still over 3 times higher than the control animals. In the iron deficient animals, significant decreases of all hematological parameters were observed during the course of the experiment, with values remaining below the control levels (Figure 9 A-D). Liver iron levels did not differ significantly from control animals during the experimental iron deficiency (Figure 9 E).

Significant and steady decreases of all hematological parameters were observed during the course of the infection (Figure 9 F-I). Liver iron levels increased slightly but not significantly from control animals (Figure 9 J).

### *Slc11a2 *expression in the *in vivo *models of iron modulation and infection

In order to determine the potential involvement of sea bass *slc11a2-α *and *slc11a2-β *in the iron metabolism and immune response, we evaluated their expression under conditions of iron modulation (overload or deficiency) or bacterial infection.

**Figure 10:**
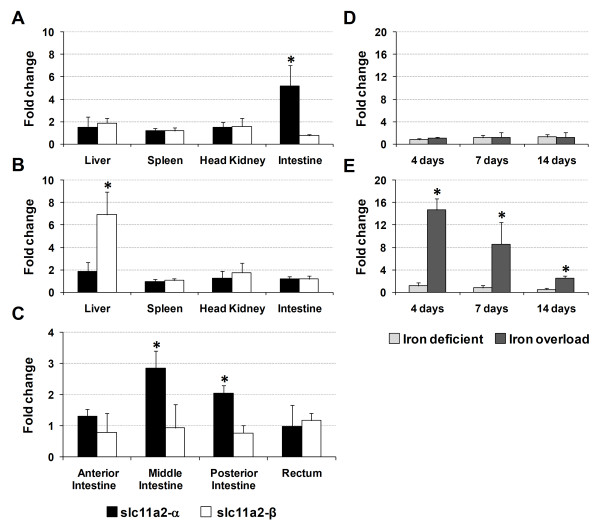
**Sea bass *slc11a2-α *and *slc11a2-β *expression in several tissues, after iron modulation**. *Slc11a2-α *and *slc11a2-β *expression 4 days after (A) iron deficiency or (B) iron overload induction; (C) *slc11a2-α *and *slc11a2-β *expression in several sections of the intestine, 4 days after iron deficiency induction; *slc11a2-α *and *slc11a2-β *expression in the liver during 14 days of (D) iron deficiency or (E) iron overload. Values are expressed as means ± S.D. (n = 5). Differences from the controls were considered significant for *p < 0.05.

In the experimental iron modulation model, *slc11a2-α *and *slc11a2-β *expressions were initially evaluated in the liver, spleen, head kidney and intestine, 4 days after iron modulation (Figure 10 A-B). In iron deficiency, there was a significant increase of *slc11a2-α *expression in the intestine, with no major changes in the other tested organs. No significant changes were observed in *slc11a2-β *expression. In iron overload, no significant changes in *slc11a2-α *were observed, whereas *slc11a2-β *expression was found to be increased in the liver. *Slc11a2-α *and *slc11a2-β *expressions were subsequently evaluated in the liver at 4, 7 and 14 days after iron modulation (Figure 10 D-E). As before, no significant changes were observed in *slc11a2-α *expression to either condition in the liver, whereas *slc11a2-β *was found t be significantly up-regulated at 4 days post iron overload (over 14-fold), decreasing during the course of the experiment to near control values, but still over 2-fold higher. Expression of *slc11a2-α *and *slc11a2-β *was also evaluated 4 days after iron modulation in several sections of the digestive tract (Figure 10 C), in order to identify the portion of the intestine responsible for the response of *slc11a2-α*. Significant increases of *slc11a2-α *expression were observed in the mid and posterior sections of the intestine, with no significant changes in the anterior section or rectum.

**Figure 11:**
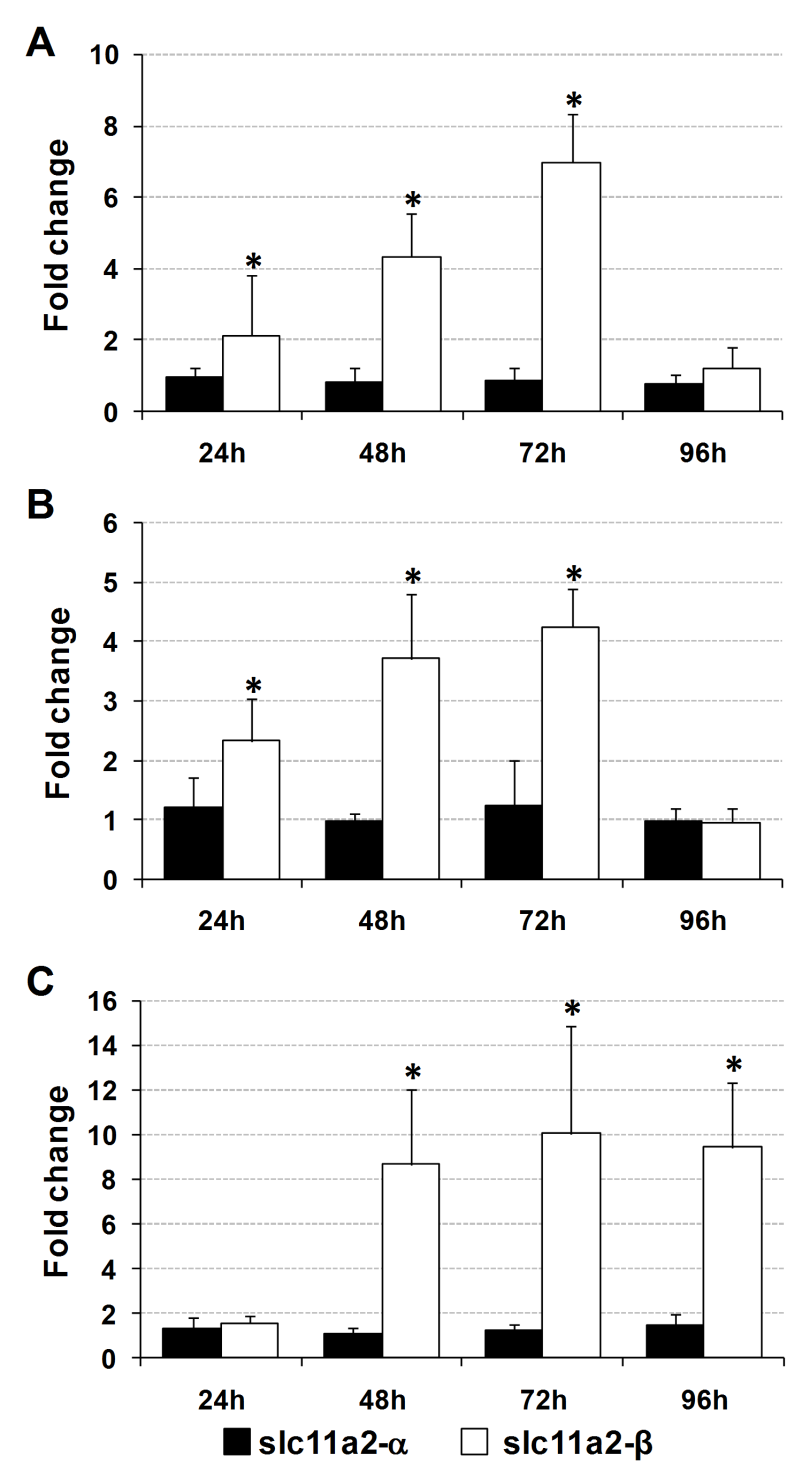
**Sea bass *slc11a2-α *and *slc11a2-β *expression under experimental infection, in the (A) liver, (B) spleen and (C) head kidney**. Values are expressed as mean fold change ± S.D. (n = 5). Samples were collected at 24, 48, 72 and 96 hours post-infection with *Photobacterium damselae *(1.0 × 10^5 ^CFU/fish). β-actin was used as the housekeeping gene. Differences from the control group were considered significant at *p < 0.05.

In the experimental model of infection, *slc11a2-α *and *slc11a2-β *expressions were measured in the liver, spleen and head kidney by real-time PCR, 24, 48, 72 and 96 h after infection. In the liver (Figure 11 A), a steady increase in *slc11a2-β *expression was observed during the course of the infection, with the maximum value at 72 h post-infection (approximately 6.8-fold increase), followed by a recovery to control levels at 96 h. In the spleen (Figure 11 B), an increase of *slc11a2-β *was also observed during the course of the infection, accompanied by a recovery to control levels at 96 h. In the head kidney (Figure 11 C), a significant increase of *slc11a2-β *was observed 24 h after infection and was maintained during its course. No significant changes were observed in *slc11a2-α *expression in any of the tested organs.

### *Slc11a2 *expression in the *in vitro *models of iron overload and infection

We investigated the role of *slc11a2-α *and *slc11a2-β *in the iron metabolism and immune response at a cellular level, by using leukocytes from spleen, one of the most important erythropoietic and immune organs in fish.

**Figure 12:**
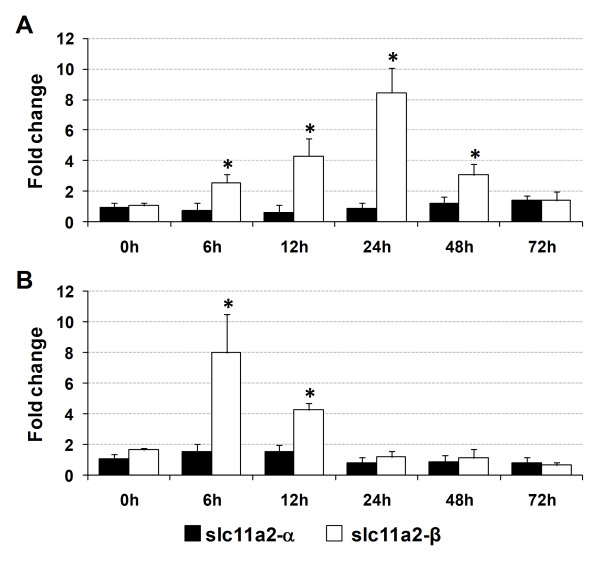
**Expression of *slc11a2-α *and *slc11a2-β *in sea bass leukocytes under experimental (a) iron overload or (b) infection, *in vitro***. Samples were collected at 0, 6, 12, 24, 48 and 72 hours after addition of ferric ammonium citrate (iron overload) or heat-inactivated *P. damselae *(infection). Values are expressed as mean fold change ± S.D. (n = 5). β-actin was used as the housekeeping gene. Differences from the control group were considered significant at *p < 0.05.

In the iron overload *in vitro *model, expression of *slc11a2-α *and *slc11a2-β *in leukocytes was measured by real-time PCR at 0, 6, 12, 24, 48 and 72 hours after the addition of ferric ammonium citrate (Figure 12 A). No significant changes were observed in *slc11a2-α *during the course of the experimental iron overload. *Slc11a2-β*, on the other hand, increased significantly from 6 h post-infection (about 2.5-fold), reaching the maximum increase at 24 h (about 8.5-fold) and decreased at 48 h, returning to control levels at 72 h.

In the *in vitro *infection model with heat-inactivated *P. damselae*, expression of *slc11a2-α *and *slc11a2-β *in leukocytes was also measured by real-time PCR at 0, 6, 12, 24, 48 and 72 hours after infection (Figure 12 B). No significant changes were observed in *slc11a2-α *during the course of infection. *Slc11a2-β*, on the other hand, increased significantly at 6 h post-infection (about 8-fold), followed by a decrease at 12 h (to about 4-fold) and returned to control levels at 24 h.

## Discussion

In the present study, we set out to analyse the evolutionary and functional patterns of *SLC11 *genes in vertebrates, in particular teleosts. We have used the European sea bass (*Dicentrarchus labrax*) as a model and have successfully isolated two *slc11 *genes, named *slc11a2-α *and *slc11a2-β*.

A single transcript was found to be produced, for the *slc11a2-α *gene, whereas *slc11a2-*β can produce up to four transcripts. The four β transcripts are the result of alternative exon splicing in the 5' and 3' ends, in a similar fashion to what has been described for human *SLC11A2 *[62,63]. But unlike human *SLC11A2*, where the differences in the 3' end result from alternate exon usage [63], in sea bass the differences result from an alternative splice site in exon 15. Thus, only the second half of exon 15 is replaced by exon 16.

Neither sea bass *slc11a2-α *nor *slc11a2-β *were found to present a potential iron responsive regulatory protein binding site (IRE) in the 3' UTR, a feature that in contrast is present in mammalian *SLC11A2 *genes [23,63] and has also been described in some teleost fish, such as fugu [36] and common carp [42]. It is, however, absent in other teleosts, such as turbot [35], channel catfish [43] and Japanese flounder [33]. In the *SLC11 *gene family, IREs have only been described in the 3'-UTR but unexpectedly, an IRE motif was found in the 5' region of *slc11a2-β*, albeit not in the 5'-UTR itself but rather 205 bp upstream the start of the 5'-UTR. The significance of this finding is still unknown, although as with other 5' IREs, it may be involved in translation repression [72,73].

The single sea bass *slc11a2-α *transcript encodes for a single functional putative protein, whereas the four *slc11a2-β *transcripts encode for four putative proteins. The four β proteins differ in the N and C termini, as a result of the encoding by the two alternative exons 1 and the alternative splice site in exon 15. Since both N and C termini are cytoplasmic, they may be involved in post-transcriptional regulatory processes and in the subcellular localization of these proteins, as occurs in mammalian SLC11A2 [74]. All sea bass Slc11a2 proteins present the characteristic features of the *SLC11 *gene family: 12 transmembrane (TM) domains, a consensus transport motif (CTM), several glycosylation and phosphorylation sites and conserved cysteine residues. Multiple alignments clearly show that these features are highly conserved and are very similar to those observed not only in other teleost fish, but also in mammals. However, there are some disparities in the features found in the 5' and 3' endings, namely some N-myristoylation and phosphorylation sites that are not present in all sea bass Slc11a2-β forms, and which may also contribute to a differential post-transcriptional regulation and subcellular localization. In all the tree models, produced by maximum-likelihood, neighbour-joining, maximum parsimony or Bayesian inference, phylogenetic analysis places all sea bass Slc11 proteins clustered with other teleost fish Slc11 proteins, which in turn cluster with mammalian SLC11A2 proteins, clearly separated from SLC11A1. Among teleost proteins, there are two clear separate clusters. One cluster encompasses sea bass Slc11a2-β protein as well as all other Slc11 and Slc11a2-β proteins. The other cluster includes sea bass Slc11a2-α and all other α proteins, with the exception of rainbow trout Slc11a2-α, suggesting that rainbow trout Slc11a2-α may have a different evolutionary origin than other Slc11a2-α proteins.

**Figure 13:**
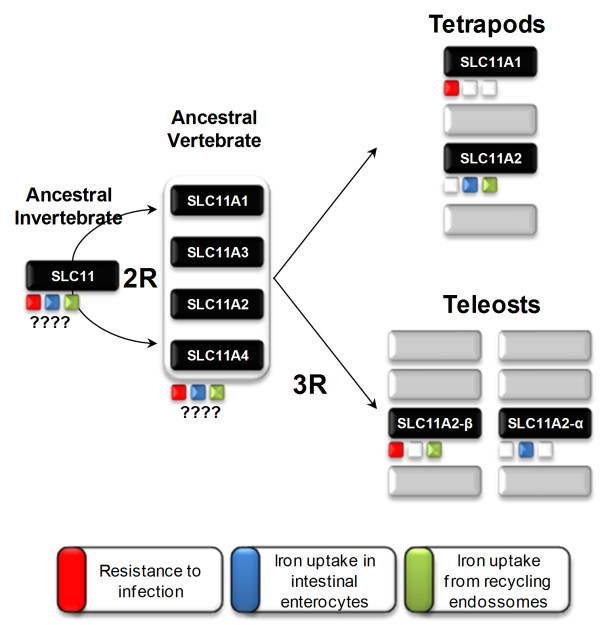
**Evolutionary model of SLC11 genes in vertebrate history**.

There are some explanations that could account for the existence of two closely related *slc11a2 *genes in sea bass, which are in agreement with what has already been proposed for other teleost fish, such as fugu. The current evolutionary scenario proposes that *slc11 *paralogs appeared through the 2R genome duplication [36,49,50], and functionally diverged. This is supported by the close linkage to HoxC and HoxD which are assumed to be duplicate *loci*. In addition, the teleost fish paralogs are more similar to mammalian SLC11A2 and may result from a third, fish specific, genome duplication [51,52]. The synteny analysis performed in the current study strongly supports the latter, suggesting that the ancestor of all vertebrates had a single *SLC11 *gene that quadrupled as part of the 2R genome duplications. Two of the new genes were lost, while two were maintained in the ancestor of tetrapods and teleosts. In teleosts the *slc11a2 *clade expanded as the result of 3R to originate two isoforms, *slc11a2-α *and *slc11a2-β*, while the *slc11a1 *ortholog was lost (Figure 13). Unlike other teleosts [36], trout's *slc11a2 *homologs do not seem to result from 3R but rather a salmonid specific tetraploidization [45].

The overall constitutive expression pattern of sea bass *slc11 *transcripts resembles that of the mammalian *SLC11A2 *gene [24-26], but also of *slc11a2 *genes described in other teleost fishes [33,35,42,43,45]. *Slc11a2-α *was found to be ubiquitously expressed, but with a higher expression along the digestive tract, with a particularly high incidence in the mid and posterior sections of the intestine. This already gives some indications for its possible involvement in intestinal iron absorption in sea bass, since the Slc11a2 mediated uptake of iron and other food derived metals in teleosts occurs predominantly in the mid and posterior regions of the intestine, as previously described [46,47]. *Slc11a2-β *was also found to be ubiquitously expressed, although unlike *slc11a2-α*, being abundant in the liver and in varying levels in all other tested tissues. No clear pattern was observed regarding the distribution of the four isoforms of *slc11a2-β*, although a prevalence of the *β1 *and *β2 *forms in most tested tissues was evident. Further analysis is needed to determine the exact function and contribution of each isoform. It is possible that they function in different subcellular compartments, with some of the forms more relevant in the immune response, while others may play a more prominent role in overall iron metabolism.

The *in situ *hybridization results in the liver, spleen, head kidney and sections of the intestine mostly reflect the measured constitutive expression. In the liver and spleen there is a clear prevalence of *slc11a2-β*, whereas in the head kidney although the constitutive expression indicates a prevalence of *slc11a2-β*, there seems to be an almost even distribution of both forms. Note that in the spleen and kidney *slc11a2-β *mRNA is clearly visible in the white pulp and lymphomyeloid tissue, respectively, which are both areas rich in B- and T-lymphocytes and actively involved in the immune response [75,76]. In the intestine, the *in situ *results show that *slc11a2-α *and *slc11a2-β *are expressed in different compartments of the enterocytes, with *slc11a2-α *mRNA mostly concentrated close to the brush border of the apical pole, whereas *slc11a2-β *is spread all over the cells, not only in the enterocytes but also in other intestinal cells. This observation provides further evidence for a sub-functionalization of *slc11a2 *homologs in sea bass, with *slc11a2-α *localization resembling that of the mammalian *SLC11A2 *IRE-positive isoforms and *slc11a2-β *with the more general distribution of mammalian *SLC11A2 *IRE-negative isoforms [62,74,77]. An issue that remains puzzling is the distribution of *slc11a2 *isoforms in the posterior intestine, with both forms concentrated in or around the goblet cells, the intestinal glandular cells that secret mucin [78]. In mammals, there are conflicting reports on the presence of *SLC11A2 *in the goblet cells, with some reporting its absence [79] while others report its presence [80], suggesting that, along with mobilferrin, *SLC11A2 *facilitates iron uptake.

To better understand the role of sea bass *slc11a2 *genes, *in vivo *and *in vitro *experimental models of iron modulation and infection were created. Haematological and iron parameters, and expression levels of *slc11a2-α *and *slc11a2-β *were evaluated under those experimental conditions.

In the experimentally iron deficient animals, with decreased haematological parameters, *slc11a2-α *expression was significantly up-regulated in the intestine, whereas *slc11a2-β *did not respond in any of the tested tissues. Further characterization of the *slc11a2-α *response to iron deficiency in the intestine showed that the up-regulation was mostly confined to the mid and posterior portions of the intestine. This is in accordance with previous findings that intestinal metal uptake in teleost fish occurs mainly in the posterior portion of the intestine [46,47], unlike mammals, where uptake occurs primarily in the duodenum [23]. These results further reinforce the idea that sea bass *slc11a2-α *seems to be mainly involved in iron uptake by the enterocytes.

In the experimentally iron overloaded animals, steady increases of the hematocrit and red blood cell counts were observed, as well as high levels of serum iron and transferrin saturation in the first days of the experiment. Also, increased levels of iron were observed in the liver, further confirming the iron overload status. *Slc11a2-α *expression did not change significantly in response to iron overload in any of the tested tissues and *slc11a2-β *was up-regulated only in the liver. Together with the increased liver iron content, this is likely associated with the need to cope with the excess of iron introduced into the system, in order to prevent its toxic effects. In normal conditions, iron in the blood is bound to transferrin, and transferrin-iron complexes are internalized by a transferrin receptor-mediated endocytic pathway. Through acidification of the endosome, iron is released from transferrin and SLC11A2 is recruited to the endosome membrane, where it exports iron to the cytoplasm. Iron is then used in cellular processes or stored in ferritin molecules. When more iron is introduced into the system, an increased expression of these genes could be expected. We have already demonstrated that during iron overload there is an increased expression of ferritin, although not accompanied by an up-regulation of transferrin, most likely due the already high levels of constitutive expression in the liver [56].

In the *in vivo *experimental infection with live *P. damselae*, a significant and continuous decrease of all haematological parameters was observed, suggesting a condition that is commonly known as anaemia of inflammation or chronic disease. This response was first described in humans [81,82] and later reported for teleost fish, namely sea bass [56,57,59] and Nile tilapia [83]. During bacterial infection, inflammatory cytokines, such as IL-6, prompt the liver to increase the production of the iron metabolism regulatory peptide hepcidin, causing the internalization of ferroportin and thus preventing cellular iron export [82,84]. The iron retention leads to impairment in erythropoiesis, due to the lack of iron availability. Overall, these changes contribute to limiting the availability of iron for pathogen growth. We have previously shown that hepcidin [59] and ferritin [56] levels are increased during bacterial infection in sea bass, thus confirming the presence of this mechanism in teleosts.

The bacterial challenge significantly up-regulated the expression of *slc11a2-β *in the liver, spleen and head kidney, in a time-dependent fashion, but produced no significant changes in *slc11a2-α *expression. A similar up-regulation was observed in *slc11a2 *homologs of other teleost fish in response to *V. anguillarum *[34,35], *Mycobacterium *[32], *E. ictaluri *[48] and LPS [43]. Further analysis will be required in order to determine whether in this case Slc11a2-β is acting as the mammalian counterpart of SLC11A1, being recruited to the membrane of pathogen-containing phagosomes, or of SLC11A2, being recruited to the membrane of holotransferrin-positive recycling endosomes involved in iron uptake from the extracellular environment. Since there are no *SLC11A1 *homologs in sea bass, and there are 4 different proteins encoded by *slc11a2-β*, it is tempting to propose that Slc11a2-β is performing both SLC11A1 and SLC11A2 mammalian functions.

In the *in vitro *iron overload of spleen-isolated leukocytes, *slc11a2-β *was found to be up-regulated in a time-dependent fashion, whereas no significant changes in *slc11a2-α *expression were observed. The increase in *slc11a2-β *expression is most likely due to the increase in iron accumulation in the macrophages, to cope with the excess iron introduced in the medium, which could become toxic for the cells.

The *in vitro *experimental infection with heat-inactivated *P. damselae*, in spleen-isolated leukocytes, produced the same effect as that observed in *vivo*, with an up-regulation of *slc11a2-β *and no changes in *slc11a2-α *expression. Not much information is available on the expression of SLC11 homologs in leukocytes, but it is known that mammalian *SLC11A1 *expression is stimulated by *M. avium *in peritoneal macrophages [85] and by LPS and IFN-γ in RAW264.7 macrophages [86]. Mammalian *SLC11A2 *expression is also stimulated by *M. avium *in peritoneal macrophages, although differentially from *SLC11A1 *[85]. Also, catfish *slc11 *was found to be stimulated in monocytes by LPS [43]. As for the *in vivo *experimental infection, it has yet to be determined whether Slc11a2-β is functioning as SLC11A1 or SLC11A2, but since both forms are known to be required for an efficient erythrophagocytosis and iron recycling in macrophages [87], it is likely that it is performing both functions. Expression studies of the four *slc11a2-β *forms and subcellular localization in the leukocytes could provide some insight on this matter.

## Conclusions

We have successfully isolated two *slc11 *paralogs in sea bass, that we named *slc11a2-α *and *slc11a2-β*. We have demonstrated that they have a role not only in iron metabolism but also in the response to bacterial infection. The clarification of the evolutionary scenario along with the functional data suggests a curious and complex pattern of sub-functionalization [88] and paralog functional equivalence, a process previously named as synfunctionalization [89,90]: the fish specific paralog *slc11a2-α *retained in part the original mammalian *SLC11A2 *function, whereas the *slc11a2-β *isoform not only retained the original *SLC11A2 *function but also acquired the *SLC11A1 *function after the loss of this isoform in teleosts (Figure 13).

## Methods

### Fish rearing

European sea bass (*Dicentrarchus labrax*), with an average weight of 50 g, were provided by a commercial fish farm in the north of Portugal (Aquacircia, Aveiro, Portugal) and reared at the fish holding facilities of the Centro Interdisciplinar de Investigação Marinha e Ambiental (CIIMAR, Porto, Portugal). Fish were maintained in 2000 liter tanks with water recirculation at a temperature of 12-14°C and constant salinity (32 ppm). The fish were fed daily to satiation with commercial fish feed (EWOS, West Lothian, UK) with an iron content of approximately 200 mg iron/kg feed and kept for more than three weeks prior to experimental use. At the beginning of each treatment fish were anaesthetized with 100 mg/l water of tricaine methanesulfonate (MS222) (Pharmaq, Fordingbridge, UK). All animal experiments were carried out in strict compliance with national and international animal use ethics guidelines, approved by the animal welfare and ethic committees of IBMC and CIIMAR (permit ref. Ofício Circular n° 99, 0420/000/000 of 09/11/2009 from the Direcção Geral de Veterinária (DGV), Portuguese Ministry of Agriculture, Rural Development and Fisheries) and conducted by FELASA Category C/DGV certified investigators.

### RNA isolation and cDNA synthesis

Fish were euthanized by anesthetic overdose of MS222 (250 mg/l), dissected and tissue samples collected, snap frozen in liquid nitrogen and stored at -80°C until further use. Total RNA was isolated with the RNeasy Midi Kit for Total RNA Isolation from Animal Cells (Qiagen, Valencia CA, USA) with the optional On-Column DNase Digestion with RNase-free DNase (Qiagen). Total RNA quantification was performed using a NanoDrop 1000 spectrophotometer (Thermo Scientific, Waltham MA, USA), quality was assessed by visualization in a denaturating formaldehyde-agarose gel and 1.25 μg of each sample were converted to cDNA by Thermoscript™ and an oligo (dT) 20 primer (Invitrogen, Carlsbad CA, USA), according to the manufacturer's protocol.

### Southern blot assay

Genomic DNA was isolated from sea bass red blood cells, as described elsewhere [91]. To determine the number of *slc11 *gene copies in sea bass, 10 μg of genomic DNA were independently digested for 24 h with EcoRI or HindII (Roche Applied Science, Mannhelm, Germany). Digestion products were run on an appropriate electrophoresis gel and blotted onto a positively charged nylon membrane (Byodine^® ^Plus Membrane, Pall Life Sciences, Ann Arbor MI, USA). The membrane was subjected to Southern analysis using the DIG System (Roche Applied Science) according to the manufacturer's specifications. Briefly, a 152 bp *slc11 *DIG labeled probe was prepared with the DIG Probe Synthesis Kit (Roche Applied Science), using primers designed based on other fish *slc11 *mRNA sequences (supplementary table S1), the membrane was hybridized at 55°C, washed, and detection was performed with the chemiluminescent substrate CDP-Star (Roche Applied Science). Visualization was achieved by exposing the membrane to X-ray film (Hyperfilm™, GE healthcare, Buckinghamshire, England) for 5-20 minutes.

### Isolation of sea bass *slc11 *genes

Several pairs of oligonucleotide PCR primers (Additional File 6, Table S1) were designed according to highly conserved regions of *slc11a1 *and *slc11a2 *mRNA sequences from other fish and mammalian species, available in the National Center for Biotechnology Information nucleotide database [92] and Ensembl [93] and cDNA preparations from liver, spleen and intestine were used in PCR amplifications. PCR products were run on 1.2% agarose gels, relevant fragments were purified with the QIAquick Gel Extraction Kit (Qiagen), cloned into pGEM-T Easy vectors, propagated in JM109 High Efficiency Competent Cells (Promega Corporation, Madison WI, USA) and sent for sequencing (Stabvida, Oeiras, Portugal).

The derived cDNA sequences were compared by megablast alignment with genomic contigs of an early version of the *D. labrax *whole genome shotgun assembly (Max Planck Institute for Molecular Genetics, Berlin, Germany). The genomic contigs were the result of an assembly of ~3× genome coverage Sanger sequencing reads and ~3× coverage 454 FLX Titanium pyro-sequencing reads by the Celera Assembler v5.3 [94]. Contigs that matched the cDNA sequences were used to build the gene models as implied by mRNA sequences.

### Rapid amplification of cDNA ends (5'and 3'RACE)

Both 5' and 3' RACE were carried out using the 5'/3' RACE Kit, 2^nd ^Generation (Roche Applied Science) according to the manufacturer's instructions. Conditions for PCR were: 94°C for 2 min, 94°C for 15 s, 60°C for 30 s, 72°C for 40 s, for 10 cycles; 94°C for 15 s, 60°C for 30 s, 72°C for 40 s (plus 20 s/cycle), for 25 cycles, with a final elongation at 72°C for 7 min. When necessary, a second PCR amplification was performed using these conditions for an additional 30 cycles. PCR products were run on 1.2% agarose gels, relevant fragments were purified with the QIAquick Gel Extraction Kit (Qiagen), cloned and sequenced as described earlier.

### Sequence analysis and alignment

Both strands of the cDNA were sequenced and assembled using Multalin [95,96] and by manual comparison of overlapping electropherograms. Alignments of the amino acid sequences of the Slc11 predicted proteins were performed using ClustalW from MEGA5 [97]. Phylogenetic tree was constructed using the Maximum-Likelihood method, with the Jones-Taylor-Thornton (JTT) model, Nearest-Neighbor-Interchange (NNI) heuristic model, complete deletion of gaps and 1000 bootstrap replications, with MEGA5 and PAUP* v4.0b10 [98]. Additional phylogenetic trees were constructed using Bayesian inference, with MrBayes v3.1.2 [99,100] and Maximum-Parsimony and Neighbor-Joining method of Saitou and Nei [101], with MEGA5. Sequences used for comparisons and phylogenetic trees and their accession numbers were as follows: from GenBank - striped bass Slc11 (AAG31225), turbot Slc11a2-β (ABB73023), turbot Slc11a2-γ (ABE97051), rainbow trout Slc11a2-α (AAD20721), rainbow trout Slc11a2-β (AAD20722), fugu Slc11a2-α (CAD43050), fugu Slc11a2-β (CAD43051), red seabream Slc11 (AAR83912), halibut Slc11 (AAX86980), channel catfish Slc11 (AAM73759), carp Slc11 (CAB60196), zebrafish Slc11 (NP_001035460), chicken SLC11A1 (NP_990295), mouse SLC11A1 (NP_038640), mouse SLC11A2 (NP_001139633), human SLC11A1 (NP_000569), human SLC11A2 -IRE (AAC21459), human SLC11A2 +IRE (NP_000608) and Drosophila Mvl-RA (NP_524425); from Ensembl - medaka Slc11a2-α (ENSORLP00000020758), medaka Slc11a2-β (ENSORLP00000019423), stickleback Slc11a2-α (ENSGACP00000015490), stickleback Slc11a2-β (ENSGACP00000000618), tetraodon Slc11a2-β (ENSTNIP00000009880), Anole lizard Slc11a1 (ENSACAP00000001836), Anole lizard Slc11a2 (ENSACAP00000012437), Xenopus Slc11a1 (ENSXETP00000032694), Xenopus Slc11a2 (ENSXETP00000021232) and *C. elegans *SMF-1 (K11G12.4b).

### Genomic organization

Genomic DNA was amplified by RT-PCR with the primers previously used for cDNA (Additional File 6, Table S1) and several PCR products were purified, cloned and sent for sequencing (Stabvida). Whole genome shotgun reads (Max Planck Institute for Molecular Genetics) were used to extend genomic regions that could not be amplified by RT-PCR. Comparisons were made between cDNA and genomic DNA to assess the similarity of the coding regions and to identify intron/exon boundaries.

Sea bass *slc11 *genes genomic organization was compared with the sequences for fugu (*Takifugu rubripes*) *slc11a2*, tetraodon (*Tetraodon *nigroviridis) *slc11a2*, xenopus (*Xenopus *tropicalis) *slc11a1 *and *slc11a2*, mouse (*Mus musculus*) *Slc11a1 *and *Slc11a2*, human (*Homo sapiens*) *SLC11A1 *and *SLC11A2 *and Drosophila (*Drosophila melanogaster*) *Mvl-RA*, available at Ensembl, release 57.

### Paralogy and synteny analysis

We identified and located the human orthologs of *SLC11A1 *(Hsa2q) and *SLC11A2 *(Hsa12q) using the Ensembl database release 57. These regions are part of the Hox paralogon [36], along with Hsa17q and Hsa3p/7. To infer if the human *SLC11A1 *and *SLC11A2 *gene environment displayed signs of duplication attributable to the 2R genome duplications, the gene content in a 1Mb Ensembl window was analysed. Gene families with more members mapping to distinct regions of the Hox paralogon were identified. Their phylogenenic relationships were inferred from the phylogenies generated by the Ensembl orthology/paralogy pipeline. Synteny data for the teleost species was retrieved from Ensembl databases. Orthology/paralogy relationships and location of human sequences were derived from the ortholog/paralog prediction function of the Ensembl website. Phylogenetic relationship of the duplicated teleost genes was determined as previously outlined.

### *Slc11a2-α *and *slc11a2-β *constitutive expression by real-time RT-PCR

Relative levels of *slc11a2-α *and *slc11a2-β *mRNAs were quantified by real-time PCR analysis using an iQ5 Multicolor Real-Time PCR Detection System (Bio-Rad, Hercules CA, USA). One μl of each cDNA sample was added to a reaction mix containing 10 μl iQ SYBR Green Supermix (Bio-Rad), 8.5 μl of _dd_H_2_0 and 250 nM of each primer, making a total volume of 20 μl per reaction. A non-template control was included for each set of primers (supplementary table S1). The cycling profile was the following: 94°C for 3.5 min, 40 cycles of 94°C for 30 s, 59°C for 30 s and 72°C for 30 s. A melting curve was generated for every PCR product to confirm the specificity of the assays and a dilution series was prepared to check the efficiency of the reactions. β-actin was used as the housekeeping gene. The comparative CT method (2^-ΔΔCT ^method) based on cycle threshold (CT) values for *slc11a2-α*, *slc11a2-β *and *β-actin *was used to analyze the expression levels of *slc11a2-α *and *slc11a2-β*.

### *Slc11a2-β *isoforms constitutive expression by semi-quantitative RT-PCR

Since the different 5' and 3' exons are separated by approximately 1.6 kb, evaluation of the constitutive expression of the four isoforms of *slc11a2-β *by real-time PCR was not a viable option. Hence, we optimized the conditions for semi-quantitative PCR to be able to reliably and specifically distinguish between these variants. To this end, exon 1A- or 1B-specific forward primers were combined with exon 15- or 16-specific reverse primers (Additional File 6, Table S1). PCRs were performed with the following conditions: 10-30 cycles of 94°C for 30 s, 59°C for 30 s and 72°C for 2 min, with an initial 5 min denaturation at 94°C and a final 10 min extension at 72°C. PCR products were resolved on ethidium bromide-stained 1.2% agarose gels, scanned on a GelDocXR+ (Biorad) and quantified using Quantity One software (Biorad).

### *In situ *hybridization

All reagents used for *in situ *hybridization were prepared with 0.1% diethyl pyrocarbonate (DEPC) in double-distilled H_2_O to rid of RNases from working solutions. Digoxigenin (DIG)-labeled anti-sense and sense riboprobes for *slc11a2-α *and *slc11a2-β *of approximately 150 bp were synthesized *in vitro *from linearized plasmid DNA, following the DIG-UTP supplier instructions (Roche Applied Science). Sections of liver, spleen, head kidney and anterior/mid/posterior regions of intestine were fixed in freshly prepared 4% paraformaldehyde in 100 mM phosphate-buffered saline (PBS; pH 7.4) at 4°C for 8 h. After dehydration, sections were embedded in paraffin, sectioned at 3 μm, mounted on poly-L-lysine coated slides, and dried at 42°C for 36 h. The sections were dewaxed in xylene 4 times (2 min each), followed by immersion in 100% ethanol (1 min), 90% ethanol (30 s), 75% ethanol (45 s) and washed with water (1 min). They were then pre-hybridized in a hybridization buffer containing 50% (v/v) deionized formamide, 50 μg/ml heparin, 5× standard saline citrate (SSC), 0.1% Tween-20, 9.2 mM citric acid pH 6.0 and 0.5 mg/ml total yeast RNA at 70°C for 2 h, and hybridized in the same hybridization buffer with 1 μg/ml of DIG-labeled anti-sense or sense riboprobes at 70°C for 12-16 h in a humidified chamber. Subsequently, the sections were subjected to several washes at 70°C: 10 min with 75% wash buffer (65% (V/V) deionized formamide, 5× SSC and 0.1% Tween-20)/25% 2× SSC, 10 min with 50% wash buffer/50% 2× SSC, 10 min with 25% wash buffer/75% 2× SSC, 10 min with 2× SSC and twice for at least 30 min with 0.05× SSC. These were followed by washes at room temperature, with shaking: 5 min in 50% 0.05× SSC/50% tris(hydroxymethyl)aminomethane-HCl (Tris-HCl) (pH 7.4) with 150 mM NaCl and twice for 5 min with 100 mM Tris-HCl (pH 7.4) with 150 mM NaCl. Slides were then pre-incubated in blocking reagent (2% normal goat serum and 2 mg/ml of bovine serum albumin) in 100 mM Tris-HCl (pH 7.4) with 150 mM NaCl for 1 h at room temperature with shaking and incubated with anti-DIG alkaline phosphatase conjugated antibody (Roche Applied Science) diluted 1:100 in blocking reagent for 2 h at room temperature. The sections were washed three times (5 min each) in 100 mM Tris-HCl (pH 7.4) with 100 mM NaCl and 50 mM MgCl_2_, then incubated with a coloring solution consisting of 4.5 μg/ml NBT and 3.5 μg/ml BCIP in 100 mM Tris-HCl (pH 8.0) with 100 mM NaCl and 50 mM MgCl_2 _for 2-24 h in the dark. The color reaction was stopped in PBS for 10 min. After rinsing in distilled water, the sections were mounted with Faramount Mounting Medium (Dako, Carpinteria CA, USA) and photographed under an Olympus BH-2 microscope (Olympus, Tokyo, Japan), with an Olympus DP25 digital camera (Olympus). Control sections were also prepared and stained with hematoxylin and eosin (H&E).

### *In vivo *model of experimental iron modulation

In order to modulate the sea bass iron status, fish reared as described before were anaesthetized and subjected to one of three different treatments, forming three experimental groups: iron overload, iron deficient and control (15 fish per group). To induce iron overload, fish were intraperitoneally injected with 200 μl of Iron Dextran (Sigma, St. Louis, MO, USA) diluted in sterile PBS to a final concentration of 10 mg/ml, as previously reported [57]. To induce the iron deficient state, fish were bled from the caudal vessels (approximately 500 μl of blood). Control fish were injected with 200 μl of sterile PBS. Four, seven and fourteen days after treatment, 5 fish from each of the experimental groups were anaesthetized and blood was drawn from the caudal vessels for evaluation of hematological parameters. Subsequently, fish were euthanized with an overdose of anesthetic, dissected and several tissues excised, snap frozen in liquid nitrogen and stored at -80°C for further iron content evaluation and gene expression analysis.

### *In vivo *model of experimental infection

*Photobacterium damselae spp. piscicida*, strain DI21, known to be pathogenic in sea bass, was used for the experimental infections. *P. damselae *was cultured to mid-logarithmic growth in tryptic soy broth (TSB) growth medium, supplemented with 1% NaCl. After measuring absorbance at 600 nm, bacteria were resuspended in TSB 1% NaCl to a final concentration of 5.0 × 10^5 ^CFUs ml^-1^.

For the experimental infection, 48 fish were anaesthetized and intraperitoneally injected with 200 μl (1.0 × 10^5 ^CFU) of bacterial suspension. For the control group, 25 fish were injected with 200 μl of TSB 1% NaCl. At 24, 48, 72 and 96 h of infection, 6 fish from each group were anaesthetized and blood drawn from the caudal vessels for evaluation of hematological parameters. Fish were then euthanized with an overdose of anesthetic, dissected and several tissues excised, snap frozen in liquid nitrogen and stored at -80°C for further iron content evaluation and gene expression analysis. Mortality was assessed every 12 h during the experimental infection.

### Hematological parameters and tissue iron in the *in vivo *experimental models

In order to determine hematological parameters, peripheral blood was drawn from the caudal vessels. For red blood cells count and hematocrit determination, 150 μl of blood were used in a 1:1 dilution with heparin in PBS (1000 units/ml). For determination of serum iron, non-heparinized blood was transferred into 1.5 ml microcentrifuge tubes, and allowed to clot for 12 h at 4°C. The samples were centrifuged twice at 16000×g until a clear serum was obtained. Serum iron (SI), unsaturated iron binding capacity (UIBC), total iron binding capacity (TIBC) and transferrin saturation (TS) were determined by the liquid ferrozine^® ^method (Thermo Electron, Victoria, Australia) according to the manufacturer's specifications.

Non-heme iron was measured in livers by the bathophenanthroline method [102]. Briefly, liver samples were weighted, placed in iron-free Teflon vessels (ACV-Advanced Composite Vessel, CEM Corporation, Matthews NC, USA) and dried in a microwave oven (MDS 2000, CEM Corporation). Subsequently, dry tissue weights were determined and samples digested in an acid mixture (30% hydrochloric acid and 10% trichloroacetic acid) for 20 h at 65°C. After digestion, a chromogen reagent (5 volumes of deionised water, 5 volumes of saturated sodium acetate and 1 volume of 0.1% bathophenanthroline sulfonate/1% thioglycollic acid) was added to the samples in order to react with iron and obtain a colored product that was measured spectrophotometrically at 535 nm. The extinction coefficient for bathophenanthroline is 22.14 mM^-1^cm^-1^.

### *In vitro *experimental models of iron overload and infection

Leucocytes were isolated from sea bass spleens. For each experiment (infection and iron overload), 5 untreated healthy fish were anesthetized and bled from the caudal vessels, then euthanized with anesthetic overdose. Spleens were aseptically dissected, placed in isolation medium (RPMI with 0.1% Fetal Bovine Serum (FBS), 1% Essential Amino Acids (A/A), 1% MEM Non-essential Amino Acid solution (MEMNEAA), 0.35% NaCl 1M and 0.4% heparin) for 10 min and macerated, with the addition of isolation medium, over a 0.4 μm mesh into a 15 ml centrifuge tube. Volumes were adjusted to 10 ml and centrifuged at 400× g for 10 min, at 4°C. The pellet was resuspended in 5 ml of isolation medium, overlaid on 5 ml of Lymphoprep (Axis-Shield PoC AS, Oslo, Norway) and centrifuged at 800×g for 30 min, at 4°C. Cells were collected from the interface and washed twice with isolation medium at 1000×g for 10 min. The pellets were resuspended in complete culture medium (RPMI with 5% FBS, 1% A/A, 1% MEMNEAA, 5% SBS and 0.35% NaCl) to a final concentration of 2.5 × 10^6 ^cells/ml.

For the iron overload experiment, cells were distributed between twelve 6-well flat bottom plates (Sarstedt, Nümbrecht, Germany), 5 fish per plate, 2 ml per well (5 × 10^6 ^cells). The control and iron overload groups (6 plates each) received 200 μl of 0.1 M ammonium citrate (Sigma) or 200 μl of 0.1 M ferric ammonium citrate (Sigma, iron content of 16.5-18.5%) per well, respectively. The latter is equivalent to approximately 8 μg of iron per well. For the experimental infection, cells were distributed as previously described and the control and infected groups (6 plates each) received 200 μl of PBS or 200 μl of heat-inactivated *Photobacterium damselae *(1 × 10^8 ^cells/ml) per well, respectively. Plates were kept in an incubator at 23°C for the duration of the experiments.

At 0, 6, 12, 24, 48 and 72 hours post-treatments, cells were collected and RNA was isolated. Briefly, cells in suspension were collected into RNase-free tubes and 0.2% trypsin in PBS was added to the wells until adherent cells detached. Complete culture medium was added and the cell suspension was transferred into the same RNase-free tubes. RNA was isolated according to the RNeasy Plus Mini protocol (Qiagen) and cDNA was prepared with Thermoscript (Invitrogen) as described before.

### *Slc11a2-α *and *slc11a2-β *expression under iron modulation and infection in the *in vivo *and *in vitro *models

The relative levels of *slc11a2-α *and *slc11a2-β *mRNAs in the organs (*in vivo *models) and leukocytes (*in vitro *models) were quantified by real-time RT-PCR. Total RNA isolation, cDNA preparation and real-time RT-PCR were performed as described before.

### Statistical analysis

Statistical analysis was carried out using PASW Statistics v17.0 for Windows (SPSS Inc., Chicago IL, USA). Data normality was checked by performing Kolmogorof-Smirnoff test and Student's T-test was used for estimating statistical significance. Multiple comparisons were performed with ANOVA. A p value less than 0.05 was considered statistically significant.

## Competing interests

The authors declare that they have no competing interests.

## Authors' contributions

JVN, JMW, PNSR conceived and designed the experiments, JVN, PNSR performed the experiments, JVN, JMW, LFCC, PNSR analysed the data, JVN, JMW, HK, RR, LFCC, PNSR contributed with reagents, materials and analysis tools, JVN, JMW, LFCC, PNSR prepared the manuscript. All authors read and approved the final manuscript.

## Supplementary Material

Additional File 1 **Figure S1: DNA and predicted amino acid sequence of sea bass slc11a2 isoforms**. This file contains the cDNA sequences, as well as the putative proteins and characteristic features for each slc11a2 isoform. Click here for file

Additional file 2 **Figure S2: Comparative view of the genomic structure, organization and size of *SLC11 *homologs of several species**. This file contains a comparative view of the genomic structure and size of sea bass *slc11a2-α *and *slc11a2-β *with homologs from other fishes, amphibians, mammals and insects. Click here for file

Additional file 3 **Figure S3: Additional phylogenetic trees**. This file contains additional phylogenetic trees constructed with Bayesian, neighbour-joining and maximum-parsimony methods. Click here for file

Additional file 4 **Figure S4: Evolutionary relationships of AGAP2, ANKRD52 and MARCH9 gene families**. This file contains phylogenetic trees for AGAP2, ANKRD52 and MARCH9, constructed with the maximum-likelihood method. Click here for file

Additional file 5 **Figure S5: Sea bass mortality during experimental infection**. This file contains a graphic showing sea bass mortality during infection with *Photobacterium damselae*, at 12 hour intervals. Click here for file

Additional file 6 **Table S1: Primers used in this study**. This file contains a table with all the primers used for sequencing, southern blot, *in situ *hybridization and gene expression analysis performed in this study. Click here for file

## References

[B1] Forbes JR Gros P Divalent-metal transport by NRAMP proteins at the interface of host-pathogen interactions Trends Microbiol 2001 9 397 403 10.1016/S0966-842X(01)02098-4 11514223

[B2] Forbes JR Gros P Iron, manganese, and cobalt transport by Nramp1 (Slc11a1) and Nramp2 (Slc11a2) expressed at the plasma membrane Blood 2003 102 1884 1892 10.1182/blood-2003-02-0425 12750164

[B3] Goswami T Bhattacharjee A Babal P Searle S Moore E Li M Blackwell JM Natural-resistance-associated macrophage protein 1 is an H+/bivalent cation antiporter Biochem J 2001 354 511 519 10.1042/0264-6021:3540511 11237855 PMC1221682

[B4] Techau ME Valdez-Taubas J Popoff JF Francis R Seaman M Blackwell JM Evolution of differences in transport function in Slc11a family members J Biol Chem 2007 282 35646 35656 10.1074/jbc.M707057200 17932044

[B5] Gruenheid S Pinner E Desjardins M Gros P Natural resistance to infection with intracellular pathogens: the Nramp1 protein is recruited to the membrane of the phagosome J Exp Med 1997 185 717 730 10.1084/jem.185.4.717 9034150 PMC2196151

[B6] Searle S Bright NA Roach TI Atkinson PG Barton CH Meloen RH Blackwell JM Localisation of Nramp1 in macrophages: modulation with activation and infection J Cell Sci 1998 111 (Pt 19) 2855 2866 9730978 10.1242/jcs.111.19.2855

[B7] Evans CA Harbuz MS Ostenfeld T Norrish A Blackwell JM Nramp1 is expressed in neurons and is associated with behavioural and immune responses to stress Neurogenetics 2001 3 69 78 10.1007/s100480100105 11354828 PMC2409433

[B8] Skamene E Schurr E Gros P Infection genomics: Nramp1 as a major determinant of natural resistance to intracellular infections Annu Rev Med 1998 49 275 287 10.1146/annurev.med.49.1.275 9509263

[B9] Vidal SM Malo D Vogan K Skamene E Gros P Natural resistance to infection with intracellular parasites: isolation of a candidate for Bcg Cell 1993 73 469 485 10.1016/0092-8674(93)90135-D 8490962

[B10] Gomes MS Appelberg R Evidence for a link between iron metabolism and Nramp1 gene function in innate resistance against Mycobacterium avium Immunology 1998 95 165 168 10.1046/j.1365-2567.1998.00630.x 9824471 PMC1364300

[B11] Abel L Sanchez FO Oberti J Thuc NV Hoa LV Lap VD Skamene E Lagrange PH Schurr E Susceptibility to leprosy is linked to the human NRAMP1 gene J Infect Dis 1998 177 133 145 10.1086/513830 9419180

[B12] Bucheton B Abel L Kheir MM Mirgani A El-Safi SH Chevillard C Dessein A Genetic control of visceral leishmaniasis in a Sudanese population: candidate gene testing indicates a linkage to the NRAMP1 region Genes Immun 2003 4 104 109 10.1038/sj.gene.6363927 12618857

[B13] Hatta M Ratnawati Tanaka M Ito J Shirakawa T Kawabata M NRAMP1/SLC11A1 gene polymorphisms and host susceptibility to Mycobacterium tuberculosis and M. leprae in South Sulawesi, Indonesia Southeast Asian J Trop Med Public Health 2010 41 386 394 20578522

[B14] Tanaka G Shojima J Matsushita I Nagai H Kurashima A Nakata K Toyota E Kobayashi N Kudo K Keicho N Pulmonary Mycobacterium avium complex infection: association with NRAMP1 polymorphisms Eur Respir J 2007 30 90 96 10.1183/09031936.00042506 17459898

[B15] Sechi LA Gazouli M Sieswerda LE Molicotti P Ahmed N Ikonomopoulos J Scanu AM Paccagnini D Zanetti S Relationship between Crohn's disease, infection with Mycobacterium avium subspecies paratuberculosis and SLC11A1 gene polymorphisms in Sardinian patients World J Gastroenterol 2006 12 7161 7164 17131479 10.3748/wjg.v12.i44.7161 PMC4087778

[B16] Esposito L Hill NJ Pritchard LE Cucca F Muxworthy C Merriman ME Wilson A Julier C Delepine M Tuomilehto J Genetic analysis of chromosome 2 in type 1 diabetes: analysis of putative loci IDDM7, IDDM12, and IDDM13 and candidate genes NRAMP1 and IA-2 and the interleukin-1 gene cluster. IMDIAB Group Diabetes 1998 47 1797 1799 10.2337/diabetes.47.11.1797 9792551

[B17] Sanjeevi CB Miller EN Dabadghao P Rumba I Shtauvere A Denisova A Clayton D Blackwell JM Polymorphism at NRAMP1 and D2S1471 loci associated with juvenile rheumatoid arthritis Arthritis Rheum 2000 43 1397 1404 10.1002/1529-0131(200006)43:6<1397::AID-ANR25>3.0.CO;2-6 10857800

[B18] Singal DP Li J Zhu Y Zhang G NRAMP1 gene polymorphisms in patients with rheumatoid arthritis Tissue Antigens 2000 55 44 47 10.1034/j.1399-0039.2000.550107.x 10703607

[B19] Gazouli M Sechi L Paccagnini D Sotgiu S Arru G Nasioulas G Vassilopoulos D NRAMP1 polymorphism and viral factors in Sardinian multiple sclerosis patients Can J Neurol Sci 2008 35 491 494 18973068 10.1017/s0317167100009173

[B20] Ates O Musellim B Ongen G Topal-Sarikaya A NRAMP1 (SLC11A1): a plausible candidate gene for systemic sclerosis (SSc) with interstitial lung involvement J Clin Immunol 2008 28 73 77 10.1007/s10875-007-9134-7 17876529

[B21] Bellamy R Susceptibility to mycobacterial infections: the importance of host genetics Genes Immun 2003 4 4 11 10.1038/sj.gene.6363915 12595896

[B22] Awomoyi AA The human solute carrier family 11 member 1 protein (SLC11A1): linking infections, autoimmunity and cancer? FEMS Immunol Med Microbiol 2007 49 324 329 10.1111/j.1574-695X.2007.00231.x 17378896 PMC1890654

[B23] Gunshin H Mackenzie B Berger UV Gunshin Y Romero MF Boron WF Nussberger S Gollan JL Hediger MA Cloning and characterization of a mammalian proton-coupled metal-ion transporter Nature 1997 388 482 488 10.1038/41343 9242408

[B24] Gruenheid S Cellier M Vidal S Gros P Identification and characterization of a second mouse Nramp gene Genomics 1995 25 514 525 10.1016/0888-7543(95)80053-O 7789986

[B25] Tabuchi M Yoshimori T Yamaguchi K Yoshida T Kishi F Human NRAMP2/DMT1, which mediates iron transport across endosomal membranes, is localized to late endosomes and lysosomes in HEp-2 cells J Biol Chem 2000 275 22220 22228 10.1074/jbc.M001478200 10751401

[B26] Vidal S Belouchi AM Cellier M Beatty B Gros P Cloning and characterization of a second human NRAMP gene on chromosome 12q13 Mamm Genome 1995 6 224 230 10.1007/BF00352405 7613023

[B27] Fleming MD Romano MA Su MA Garrick LM Garrick MD Andrews NC Nramp2 is mutated in the anemic Belgrade (b) rat: evidence of a role for Nramp2 in endosomal iron transport Proc Natl Acad Sci USA 1998 95 1148 1153 10.1073/pnas.95.3.1148 9448300 PMC18702

[B28] Fleming MD Trenor CC Su MA Foernzler D Beier DR Dietrich WF Andrews NC Microcytic anaemia mice have a mutation in Nramp2, a candidate iron transporter gene Nat Genet 1997 16 383 386 9241278 10.1038/ng0897-383

[B29] Cellier M Govoni G Vidal S Kwan T Groulx N Liu J Sanchez F Skamene E Schurr E Gros P Human natural resistance-associated macrophage protein: cDNA cloning, chromosomal mapping, genomic organization, and tissue-specific expression J Exp Med 1994 180 1741 1752 10.1084/jem.180.5.1741 7964458 PMC2191750

[B30] Kishi F Isolation and characterization of human Nramp cDNA Biochem Biophys Res Commun 1994 204 1074 1080 10.1006/bbrc.1994.2572 7980580

[B31] Hu J Bumstead N Burke D Ponce de Leon FA Skamene E Gros P Malo D Genetic and physical mapping of the natural resistance-associated macrophage protein 1 (NRAMP1) in chicken Mamm Genome 1995 6 809 815 10.1007/BF00539010 8597640

[B32] Burge EJ Gauthier DT Ottinger CA Van Veld PA Mycobacterium-inducible Nramp in striped bass (Morone saxatilis) Infect Immun 2004 72 1626 1636 10.1128/IAI.72.3.1626-1636.2004 14977970 PMC356044

[B33] Chen SL Wang ZJ Xu MY Gui JF Molecular identification and expression analysis of natural resistance associated macrophage protein (Nramp) cDNA from Japanese flounder (Paralichthys olivaceus) Fish Shellfish Immunol 2006 20 365 373 10.1016/j.fsi.2005.05.011 15998592

[B34] Chen SL Xu MY Ji XS Yu GC Cloning and characterisation of natural resistance associated macrophage protein (Nramp) cDNA from red sea bream (Pagrus major) Fish Shellfish Immunol 2004 17 305 313 10.1016/j.fsi.2004.04.003 15312657

[B35] Chen SL Zhang YX Xu JY Meng L Sha ZX Ren GC Molecular cloning, characterization and expression analysis of natural resistance associated macrophage protein (Nramp) cDNA from turbot (Scophthalmus maximus) Comp Biochem Physiol B Biochem Mol Biol 2007 147 29 37 10.1016/j.cbpb.2006.12.003 17317252

[B36] Sibthorpe D Baker AM Gilmartin BJ Blackwell JM White JK Comparative analysis of two slc11 (Nramp) loci in Takifugu rubripes DNA Cell Biol 2004 23 45 58 10.1089/104454904322745925 14965472

[B37] Rodrigues V Cheah PY Ray K Chia W malvolio, the Drosophila homologue of mouse NRAMP-1 (Bcg), is expressed in macrophages and in the nervous system and is required for normal taste behaviour EMBO J 1995 14 3007 3020 7621816 10.1002/j.1460-2075.1995.tb07303.x PMC394361

[B38] Consortium TCeS Genome Sequence of the Nematode C. elegans: A Platform for Investigating Biology Science 1998 282 2012 2018 10.1126/science.282.5396.2012 9851916

[B39] Belouchi A Cellier M Kwan T Saini HS Leroux G Gros P The macrophage-specific membrane protein Nramp controlling natural resistance to infections in mice has homologues expressed in the root system of plants Plant Mol Biol 1995 29 1181 1196 10.1007/BF00020461 8616217

[B40] Portnoy ME Liu XF Culotta VC Saccharomyces cerevisiae expresses three functionally distinct homologues of the nramp family of metal transporters Mol Cell Biol 2000 20 7893 7902 10.1128/MCB.20.21.7893-7902.2000 11027260 PMC86400

[B41] Makui H Roig E Cole ST Helmann JD Gros P Cellier MF Identification of the Escherichia coli K-12 Nramp orthologue (MntH) as a selective divalent metal ion transporter Mol Microbiol 2000 35 1065 1078 10.1046/j.1365-2958.2000.01774.x 10712688

[B42] Saeij JP Wiegertjes GF Stet RJ Identification and characterization of a fish natural resistance-associated macrophage protein (NRAMP) cDNA Immunogenetics 1999 50 60 66 10.1007/s002510050686 10541807

[B43] Chen H Waldbieser GC Rice CD Elibol B Wolters WR Hanson LA Isolation and characterization of channel catfish natural resistance associated macrophage protein gene Dev Comp Immunol 2002 26 517 531 10.1016/S0145-305X(01)00096-9 12031412

[B44] Donovan A Brownlie A Dorschner MO Zhou Y Pratt SJ Paw BH Phillips RB Thisse C Thisse B Zon LI The zebrafish mutant gene chardonnay (cdy) encodes divalent metal transporter 1 (DMT1) Blood 2002 100 4655 4659 10.1182/blood-2002-04-1169 12393445

[B45] Dorschner MO Phillips RB Comparative analysis of two Nramp loci from rainbow trout DNA Cell Biol 1999 18 573 583 10.1089/104454999315123 10433556

[B46] Bury N Grosell M Iron acquisition by teleost fish Comp Biochem Physiol C Toxicol Pharmacol 2003 135 97 105 10.1016/S1532-0456(03)00021-8 12860048

[B47] Bury NR Walker PA Glover CN Nutritive metal uptake in teleost fish J Exp Biol 2003 206 11 23 10.1242/jeb.00068 12456693

[B48] Elibol-Flemming B Waldbieser GC Wolters WR Boyle CR Hanson LA Expression analysis of selected immune-relevant genes in channel catfish during Edwardsiella ictaluri infection J Aquat Anim Health 2009 21 23 35 10.1577/H08-009.1 19485123

[B49] Holland PW Garcia-Fernandez J Williams NA Sidow A Gene duplications and the origins of vertebrate development Dev Suppl 1994 125 133 7579513

[B50] Putnam NH Butts T Ferrier DE Furlong RF Hellsten U Kawashima T Robinson-Rechavi M Shoguchi E Terry A Yu JK The amphioxus genome and the evolution of the chordate karyotype Nature 2008 453 1064 1071 10.1038/nature06967 18563158

[B51] Taylor JS Van de Peer Y Braasch I Meyer A Comparative genomics provides evidence for an ancient genome duplication event in fish Philos Trans R Soc Lond B Biol Sci 2001 356 1661 1679 10.1098/rstb.2001.0975 11604130 PMC1088543

[B52] Taylor JS Van de Peer Y Meyer A Revisiting recent challenges to the ancient fish-specific genome duplication hypothesis Curr Biol 2001 11 R1005 1008 10.1016/S0960-9822(01)00610-8 11747834

[B53] Chistiakov DA Hellemans B Volckaert FA Review on the immunology of European sea bass Dicentrarchus labrax Vet Immunol Immunopathol 2007 117 1 16 10.1016/j.vetimm.2007.02.005 17382407

[B54] Kuhl H Beck A Wozniak G Canario AV Volckaert FA Reinhardt R The European sea bass Dicentrarchus labrax genome puzzle: comparative BAC-mapping and low coverage shotgun sequencing BMC Genomics 2010 11 68 10.1186/1471-2164-11-68 20105308 PMC2837037

[B55] Negrisolo E Kuhl H Forcato C Vitulo N Reinhardt R Patarnello T Bargelloni L Different Phylogenomic Approaches to Resolve the Evolutionary Relationships among Model Fish Species Mol Biol Evol 2010 27 2757 2774 10.1093/molbev/msq165 20591844

[B56] Neves JV Wilson JM Rodrigues PN Transferrin and ferritin response to bacterial infection: the role of the liver and brain in fish Dev Comp Immunol 2009 33 848 857 10.1016/j.dci.2009.02.001 19428486

[B57] Rodrigues PN Pereira FA A model for acute iron overload in sea bass (Dicentrarchus labrax L.) Lab Anim 2004 38 418 424 10.1258/0023677041958909 15479557

[B58] Rodrigues PN Pereira FA Effect of dietary iron overload on Photobacterium damselae ssp. piscicida pathogenicity in sea bass, Dicentrarchus labrax (L.) J Fish Dis 2004 27 673 676 10.1111/j.1365-2761.2004.00580.x 15509262

[B59] Rodrigues PN Vazquez-Dorado S Neves JV Wilson JM Dual function of fish hepcidin: response to experimental iron overload and bacterial infection in sea bass (Dicentrarchus labrax) Dev Comp Immunol 2006 30 1156 1167 10.1016/j.dci.2006.02.005 16616368

[B60] Zanuy S Carrillo M Felip A Rodríguez L Blázquez M Ramos J Piferrer F Genetic, hormonal and environmental approaches for the control of reproduction in the European sea bass (Dicentrarchus labrax L.) Aquaculture 2001 202 187 203 10.1016/S0044-8486(01)00771-2

[B61] Wiegertjes GF Stet RJ Parmentier HK van Muiswinkel WB Immunogenetics of disease resistance in fish: a comparative approach Dev Comp Immunol 1996 20 365 381 10.1016/S0145-305X(96)00032-8 9040980

[B62] Hubert N Hentze MW Previously uncharacterized isoforms of divalent metal transporter (DMT)-1: implications for regulation and cellular function Proc Natl Acad Sci USA 2002 99 12345 12350 10.1073/pnas.192423399 12209011 PMC129447

[B63] Lee PL Gelbart T West C Halloran C Beutler E The human Nramp2 gene: characterization of the gene structure, alternative splicing, promoter region and polymorphisms Blood Cells Mol Dis 1998 24 199 215 10.1006/bcmd.1998.0186 9642100

[B64] Alternative Splice Site Predictor http://www.es.embnet.org/~mwang/assp.html

[B65] Wang M Marin A Characterization and prediction of alternative splice sites Gene 2006 366 219 227 10.1016/j.gene.2005.07.015 16226402

[B66] RegRNA http://regrna.mbc.nctu.edu.tw/index.php

[B67] Huang HY Chien CH Jen KH Huang HD RegRNA: an integrated web server for identifying regulatory RNA motifs and elements Nucleic Acids Res 2006 34 W429 434 10.1093/nar/gkl333 16845041 PMC1538840

[B68] ExPASy http://www.expasy.org/tools/

[B69] Cellier M Prive G Belouchi A Kwan T Rodrigues V Chia W Gros P Nramp defines a family of membrane proteins Proc Natl Acad Sci USA 1995 92 10089 10093 10.1073/pnas.92.22.10089 7479731 PMC40741

[B70] Dreborg S Sundstrom G Larsson TA Larhammar D Evolution of vertebrate opioid receptors Proc Natl Acad Sci USA 2008 105 15487 15492 10.1073/pnas.0805590105 18832151 PMC2563095

[B71] Jaillon O Aury JM Brunet F Petit JL Stange-Thomann N Mauceli E Bouneau L Fischer C Ozouf-Costaz C Bernot A Genome duplication in the teleost fish Tetraodon nigroviridis reveals the early vertebrate proto-karyotype Nature 2004 431 946 957 10.1038/nature03025 15496914

[B72] Eisenstein RS Iron regulatory proteins and the molecular control of mammalian iron metabolism Annu Rev Nutr 2000 20 627 662 10.1146/annurev.nutr.20.1.627 10940348

[B73] Hentze MW Muckenthaler MU Andrews NC Balancing acts: molecular control of mammalian iron metabolism Cell 2004 117 285 297 10.1016/S0092-8674(04)00343-5 15109490

[B74] Tabuchi M Tanaka N Nishida-Kitayama J Ohno H Kishi F Alternative splicing regulates the subcellular localization of divalent metal transporter 1 isoforms Mol Biol Cell 2002 13 4371 4387 10.1091/mbc.E02-03-0165 12475959 PMC138640

[B75] Cesta MF Normal structure, function, and histology of the spleen Toxicol Pathol 2006 34 455 465 10.1080/01926230600867743 17067939

[B76] Zapata AG Chibá A Varas A George I, Teruyuki N 1 Cells and Tissues of the Immune System of Fish Fish Physiology. Volume 1997 15 Academic Press 1 62 full_text

[B77] Lam-Yuk-Tseung S Gros P Distinct targeting and recycling properties of two isoforms of the iron transporter DMT1 (NRAMP2, Slc11A2) Biochemistry 2006 45 2294 2301 10.1021/bi052307m 16475818

[B78] Specian RD Oliver MG Functional biology of intestinal goblet cells Am J Physiol 1991 260 C183 193 1996606 10.1152/ajpcell.1991.260.2.C183

[B79] Canonne-Hergaux F Gruenheid S Ponka P Gros P Cellular and subcellular localization of the Nramp2 iron transporter in the intestinal brush border and regulation by dietary iron Blood 1999 93 4406 4417 10361139

[B80] Conrad ME Umbreit JN Pathways of iron absorption Blood Cells Mol Dis 2002 29 336 355 10.1006/bcmd.2002.0564 12547224

[B81] Cartwright GE Wintrobe MM The anemia of infection. XVII. A review Adv Intern Med 1952 5 165 226 12996335

[B82] Andrews NC Anemia of inflammation: the cytokine-hepcidin link J Clin Invest 2004 113 1251 1253 15124013 10.1172/JCI21441 PMC398435

[B83] Chen CY Wooster GA Bowser PR Comparative blood chemistry and histopathology of tilapia infected with Vibrio vulnificus or Streptococcus iniae or exposed to carbon tetrachloride, gentamicin, or copper sulfate Aquaculture 2004 239 421 443 10.1016/j.aquaculture.2004.05.033

[B84] Nemeth E Rivera S Gabayan V Keller C Taudorf S Pedersen BK Ganz T IL-6 mediates hypoferremia of inflammation by inducing the synthesis of the iron regulatory hormone hepcidin J Clin Invest 2004 113 1271 1276 15124018 10.1172/JCI20945 PMC398432

[B85] Zhong W Lafuse WP Zwilling BS Infection with Mycobacterium avium differentially regulates the expression of iron transport protein mRNA in murine peritoneal macrophages Infect Immun 2001 69 6618 6624 10.1128/IAI.69.11.6618-6624.2001 11598030 PMC100035

[B86] Govoni G Canonne-Hergaux F Pfeifer CG Marcus SL Mills SD Hackam DJ Grinstein S Malo D Finlay BB Gros P Functional expression of Nramp1 in vitro in the murine macrophage line RAW264.7 Infect Immun 1999 67 2225 2232 10225878 10.1128/iai.67.5.2225-2232.1999 PMC115961

[B87] Soe-Lin S Apte SS Mikhael MR Kayembe LK Nie G Ponka P Both Nramp1 and DMT1 are necessary for efficient macrophage iron recycling Exp Hematol 2010 38 609 617 10.1016/j.exphem.2010.04.003 20394798

[B88] Force A Lynch M Pickett FB Amores A Yan YL Postlethwait J Preservation of duplicate genes by complementary, degenerative mutations Genetics 1999 151 1531 1545 10101175 10.1093/genetics/151.4.1531 PMC1460548

[B89] Gitelman I Evolution of the vertebrate twist family and synfunctionalization: a mechanism for differential gene loss through merging of expression domains Mol Biol Evol 2007 24 1912 1925 10.1093/molbev/msm120 17567594

[B90] Liberles DA Gene duplication: red queens, linkage, redundancy and synfunctionalization Heredity 2008 101 299 300 10.1038/hdy.2008.81 18698335

[B91] Cummings SA Thorgaard GH Extraction of DNA from fish blood and sperm Biotechniques 1994 17 426, 428, 430 7818889

[B92] National Center for Biotechnology Information http://www.ncbi.nlm.nih.gov

[B93] Ensembl http://www.ensembl.org

[B94] Miller JR Delcher AL Koren S Venter E Walenz BP Brownley A Johnson J Li K Mobarry C Sutton G Aggressive assembly of pyrosequencing reads with mates Bioinformatics 2008 24 2818 2824 10.1093/bioinformatics/btn548 18952627 PMC2639302

[B95] Multalin http://bioinfo.genopole-toulouse.prd.fr/multalin/multalin.html

[B96] Corpet F Multiple sequence alignment with hierarchical clustering Nucleic Acids Res 1988 16 10881 10890 10.1093/nar/16.22.10881 2849754 PMC338945

[B97] Tamura K Peterson D Peterson N Stecher G Nei M Kumar S MEGA5: Molecular Evolutionary Genetics Analysis using Maximum Likelihood, Evolutionary Distance, and Maximum Parsimony Methods Mol Biol Evol 2011 (submitted) 10.1093/molbev/msr121 PMC3203626 21546353

[B98] Swofford DL PAUP*. Phylogenetic Analysis Using Parsimony (*and Other Methods). Version 4 2003 Sunderland, Massachusetts: Sinauer Associates

[B99] Ronquist F Huelsenbeck JP MrBayes 3: Bayesian phylogenetic inference under mixed models Bioinformatics 2003 19 1572 1574 10.1093/bioinformatics/btg180 12912839

[B100] Huelsenbeck JP Ronquist F MRBAYES: Bayesian inference of phylogenetic trees Bioinformatics 2001 17 754 755 10.1093/bioinformatics/17.8.754 11524383

[B101] Saitou N Nei M The neighbor-joining method: a new method for reconstructing phylogenetic trees Mol Biol Evol 1987 4 406 425 3447015 10.1093/oxfordjournals.molbev.a040454

[B102] Torrence JD Bothwell TH Cook JD Tissue Iron Stores Methods in haematology 1980 New York: Churchill Livingston Press 104 109 [JD C (Series Editor)

