# Supplementary material for: Natural history of SLC11 genes in vertebrates: tales from the fish world
Source: BMC Evol Biol. 2011 Apr 18;11:106. doi: 10.1186/1471-2148-11-106 (PMC3103463; doi:10.1186/1471-2148-11-106)
Supplement: Additional File 1 — Figure S1: DNA and predicted amino acid sequence of sea bass slc11a2 isoforms. This file contains the cDNA sequences, as well as the putative proteins and characteristic features for each slc11a2 isoform. [file 1471-2148-11-106-S1.DOC]

**Additional File 1, Figure S1 – DNA and predicted amino acid sequence of sea bass (A) *scl11a2-α*, (B) *slc11a2-β1*, (C) *slc11a2-β2*, (D) *slc11a2-β3* and (E) *slc11a2-β4***. Nucleotides are indicated above and numbered to the right of each lane (upper row). The deducted amino acid is shown below the nucleotide sequence. Amino acids are indicated with italic letters and numbered to the right of each lane (lower row) ending with the TAG stop codon (represented by *). The transmembrane regions (TM) are underlined with and numbered 1-12. The consensus transport motif (CTM) located between TM8 and TM9 is underlined with . Protein kinase C phosphorylation sites (S/T-X-R/K) are underlined with , N-linked glycosylation sites (N-X-S/T-X) are underlined with , casein kinase II phosphorylation sites (S/T-XX-D/E) are underlined with oo o o, tyrosine kinase phosphorylation sites (R/K-XX(orXXX)-D/E-XX(orXXX)-Y) are underlined with , tyrosine based sorting signal (NPXY or YXXΦ; where Φ is a bulky hydrophobic residue) is underlined with oo o o and conserved cysteine residues are boxed *C*.

**A**

| ACGTAATAGTAATCGCTGATATTGCC | 26 |
| --- | --- |
| CAAACTTTTTCCCTTGAATGACTGAAAACTGACACTTAATGCTGGGCCGCGGGCCAAAAGCAGACACCTGTTGTATTGTTAAGATGCAAA  ATGGCACCAGGAACTTCTTGCGCAAGATGTCAGGCAGATGACCTGGTGGAAGAGAAAGCAGTGATCCAGACCACACACACACACAGAGTC | 116  206 |
| *M A P G T S C A R C Q A D D L V E E K A V I Q T T H T H R V* | 30 |
| TCGGCCCCCTCGGTCTTCCTCCAGAAACACAATAATGAACCTGTTTCCAGCACTTACTTCGACCAGAGGGTCCCCGTTCCCGAGGAGGAC | 2960 |
| *S A P S V F L Q K H N N E P V S S T Y F D Q R V P V P E E D* | 60 |
| AGCGAGTGGGGGATTAGTCTCCGTAAACTGTGGGCCTTCACTGGGCCGGGGTTTCTGATGAGCATAGCCTACCTGGACCCAGGTAACATC | 3860 |
| *S E W G I S L R K L W A F T G P G F L M S I A Y L D P G N I* | 90 |
| TM 1 |  |
| GAGTCTGACCTGCAGTCTGGTGCTAAAGCTGGCTTCAAGCTCCTCTGGGTGCTGCTGGGAGCCACCATCATCGGCCTGCTGCTGCAGCGT | 4760 |
| *E S D L Q S G A K A G F K L L W V L L G A T I I G L L L Q R* | 120 |
| TM 2 |  |
| CTGGCGGCTCGGCTGGGCGTCGTCACCGGGATGCACCTGGCTGAAGTCTGCAACCGCCAGTACCGCACTGTGCCTCGTGTCATCCTGTGG | 5660 |
| *L A A R L G V V T G M H L A E V C N R Q Y R T V P R V I L W* | 150 |
| TTGATGGTGGAGCTGGCGATCATTGGCTCAGACATGCAGGAGGTCATCGGCTGTGCCATCGCGTTCAACCTCCTCTCCTCTGGCAGGATC | 6560 |
| *L M V E L A I I G S D M Q E V I G C A I A F N L L S S G R I*  TM 3 | 180 |
| CCGTTGTGGGCTGGCGTCCTCATCACCATCATCGACACCTTTGTCTTCCTCTTCTTGGACAAGTATGGCCTGAGGAAGCTGGAAGCCTTC | 7460 |
| *P L W A G V L I T I I D T F V F L F L D K Y G L R K L E A F* | 210 |
| TM 4 |  |
| TTTGGTGTGCTCATCACCATCATGGCCATCACCTTTGGATATGAGTATGTGACGGTGAGGCCAGACCAAGGCGAGCTGCTGAAGGGGATG | 8360 |
| *F G V L I T I M A I T F G Y E Y V T V R P D Q G E L L K G M*  TM 5 | 240 |
| TTTGTGCCGTACTGCGAGGGCTGCGGGCCCGTTCAGCTGGCTCAGGCCGTCGGCATCGTGGGAGCCGTCATCATGCCTCACAACATTTAC | 926 |
| *F V P Y C E G C G P V Q L A Q A V G I V G A V I M P H N I Y* | 270 |
| TM 6 |  |
| CTCCACTCTGCTCTTGTCAAGTCTCGAGAAGTGGATCGGTCGAACAGGAAAGAAGTCAAAGAGGCCAACAAATACTTCTTCATTGAGGCG | 1016 |
| L H S A L V K S R E V D R S N R K E V K E A N K Y F F I E A | 300 |
| ACCATCGCGCTGTTCGTCTCGTTCCTCATCAACGTGTTTGTGGTGGCTGTTTTCGCCGAGGCTTTCTACGGACGCACAAACGAAGAGGTG | 1106 |
| T I A L F V S F L I N V F V V A V F A E A F Y G R T N E E V | 330 |
| TM 7 |  |
| CACACTGTCTGCAATCAATCAGGAAGTCCTCATTCATCTCTGTTCCCTCTGAACAATGAAACTCTAGAGGTGGACATCTACAAAGGGGGA | 1196 |
| H T V C N Q S G S P H S S L F P L N N E T L E V D I Y K G G | 360 |
| GTGGTGCTCGGCTGCTTCTTTGGCCCGGCCGCGCTCTACATCTGGGCCGTGGGGATCCTTGCAGCTGGTCAGAGCTCCACCATGACCGGC | 1286 |
| V V L G C F F G P A A L Y I W A V G I L A A G Q S S T M T G | 390 |
| TM 8 |  |
| ACGTACTCTGGTCAGTTCGTCATGGAGGGTTTCTTGAACCTGCGCTGGTCGCGCTTCGCTCGGGTGTTGCTGACCCGCTCCCTCGCCATC | 1376 |
| T Y S G Q F V M E G F L N L R W S R F A R V L L T R S L A I | 420 |
| CTM TM 9 |  |
| ACTCCCACCCTGCTGGTTGCCATCTTCCAGGACGTGCAGCACCTGACGGGCATGACCGACTTCCTCAATGTGCTGCAGAGCATGCAGTTG | 1466 |
| T P T L L V A I F Q D V Q H L T G M T D F L N V L Q S M Q L | 450 |
| TM 10 |  |
| CCATTTGCCCTCATTCCCATCCTCACGTTCACCAGTTTGCCGTCTCTCATGAATGAGTTTGCCAACGGATTGGGGTCTAAAATCGGAGGA | 1556 |
| P F A L I P I L T F T S L P S L M N E F A N G L G S K I G G | 480 |
| GGGCTGGTGATCTTGCTCGTGTGTGCCATCAACATGTACTTCGTCGTGGTCTACGTGACAACATTCAACAGTGTGTGGCTGTATGTGCTG | 1646 |
| G L V I L L V C A I N M Y F V V V Y V T T F N S V W L Y V L | 510 |
| TM 11 |  |
| GCAACGTTCTTCTCCGTAGCATACCTGACATTCGTGGGATATCTGGTGTGGTTGTGTCTGATAGCTCTGGGAGTGTCCTGCCTCGATCCG | 1736 |
| A T F F S V A Y L T F V G Y L V W L C L I A L G V S C L D P | 540 |
| TM 12 |  |
| TCTTCCAAGAGGGCCAACGACACGACCGTCCTCATCGAACGGCAGCCGGAGTTCGACTCCTGAATCCAACGTGTGTTTGAGTGGACGTTT | 1826 |
| S S K R A N D T T V L I E R Q P E F D S *  TAAAGGTGCAGCTGTGACTGACCCTTTTGTTTAAAGTCACACTCTAATGATTATACAAAACTAGTATGGACAAAAAAAAAAAAAAAAAAA  AAA | 560  1916  1919 |

**B**

| TTCCTTCTTGTGCTTATTATGAG  AAAGCATGAAGTTTCTATACAAAACTAATCTTTTTAGCTCAATACTTGCATGATAATTACTGTTTTACCCTCAACAGACATGTCTTTCAT | 23  1130 |
| --- | --- |
| GCTCCTTCAGATTATGTAATTGTTGTTTTCTTATTCCAGGGAGGCTGATATTCATCCCTCAAGTTCCCAGGGATACCTCAGCTTGCCCTG  ATGAAAGCCGAGCAAGACGGAGACCTCCTCGAAATGAAGGAAGGAAAGAATGAAAAGTCCACTCTCAAGGACTCTCCTCAGGACAATGGA | 203  293 |
| *M K A E Q D G D L L E E D S P Q D N G V Q T N Q Y S S I S P* | 30 |
| CCAGCCTCACCTGTGGCCCAGGAGGAACCTTTTTCTACATACTTTGAAGACAAGGTTGCCATTCCCGAAAATGTCAACCAGGTATTCAGT | 3830 |
| *P A S P V A Q E E P F S T Y F E D K V A I P E N V N Q V F S* | 60 |
| TTCCGAAAACTCTGGGCATTCACTGGACCAGGGTTTTTGATGAGCATTGCTTACTTGGATCCAGGAAACATCGAGTCTGACCTGCAGTCT | 4730 |
| *F R K L W A F T G P G F L M S I A Y L D P G N I E S D L Q S* | 90 |
| TM 1 |  |
| GGAGCTAAAGCAGGCTTTAAGCTCCTGTGGGTGCTACTTGTGGCCACCATCATTGGGCTGCTGTTGCAGAGGTTAGCTGCACGCCTTGGT | 5630 |
| *G A K A G F K L L W V L L V A T I I G L L L Q R L A A R L G* | 120 |
| TM 2 |  |
| GTCGTCACTGGGATGCACCTGGCTGAAGTCTGCAACCGCCAATATCCCACAGTTCCTCGGGTCATCCTTTGGCTGATGGTGGAACTGGCA | 6530 |
| *V V T G M H L A E V C N R Q Y P T V P R V I L W L M V E L A* | 150 |
| ATTATTGGCTCAGACATGCAGGAGGTCATTGGCTGTGCCATTGCTCTCAACCTTCTCTCTGTGGGAAGGATTCCACTGTGGGCAGGAGTC | 7430 |
| *I I G S D M Q E V I G C A I A L N L L S V G R I P L W A G V*  TM 3 | 180 |
| CTCATCACCATCACAGACACATTCGTGTTCCTTTTTCTAGACAAATACGGCCTGAGGAAACTTGAAGCTTTCTTTGGATTTCTCATCACT | 8330 |
| *L I T I T D T F V F L F L D K Y G L R K L E A F F G F L I T* | 210 |
| TM 4 |  |
| GTAATGGCTCTTAGCTTCGGTTACGAGTATGTGCTGGTAAAGCCAGATCAAGGGGAACTTCTGAAGGGGATGTTCTTACCTTACTGCGCC | 9230 |
| *V M A L S F G Y E Y V L V K P D Q G E L L K G M F L P Y C A*  TM 5 | 240 |
| GGCTGTGGGCCTGTGCAGCTGGAGCAGGCAGTGGGCATCGTAGGTGCTGTCATCATGCCCCACAACATCTACTTGCACTCAGCGCTGGTC | 1013 |
| *G C G P V Q L E Q A V G I V G A V I M P H N I Y L H S A L V* | 270 |
| TM 6 |  |
| AAGTCTCGAGACATCGATCGGAAAAATAAGAAGGAAGTAAAGGAAGCCAACAAGTACTACTTCATTGAGTCAACTATTGCTCTCTTTATC | 1103 |
| *K S R D I D R K N K K E V K E A N K Y Y F I E S T I A L F I* | 300 |
| TCCTTCCTCATCAACGTCTTTGTCGTAGCGGTCTTTGCTGAGGCCTTCTACAATAAAACCAATATGGAAGTGCATGAATTTTGCAATAAA | 1193 |
| *S F L I N V F V V A V F A E A F Y N K T N M E V H E F C N K* | 330 |
| TM 7 |  |
| ACTGGCAGCCCTCACTCAGGTCTTTTCCCACAAGACAACAACACACTGGAGGTGGACATCTACAAAGGGGGAGTGGTTCTGGGCTGTGTC | 1283 |
| *T G S P H S G L F P Q D N N T L E V D I Y K G G V V L G C V* | 360 |
| TTTGGCCCTGCAGCCCTCTACATCTGGGCAATAGGGATCCTGGCAGCTGGACAGAGTTCCACCATGACAGGCACTTACTCTGGGCAGTTT | 1373 |
| *F G P A A L Y I W A I G I L A A G Q S S T M T G T Y S G Q F* | 390 |
| TM 8 CTM |  |
| GTTATGGAGGGTTTCCTGAACCTACGGTGGTCCCGTTTTGCTCGGGTGCTGCTGACCCGCTCCATCGCCATCACGCCAACACTGCTGGTA | 1463 |
| *V M E G F L N L R W S R F A R V L L T R S I A I T P T L L V* | 420 |
| TM 9 |  |
| GCCATTTTCCAGGATGTTCAGCATCTGACAGGGATGAACGACTTCCTCAACGTGCTTCAAAGCATGCAGCTTCCGTTTGCTTTGATACCA | 1553 |
| *A I F Q D V Q H L T G M N D F L N V L Q S M Q L P F A L I P* | 450 |
| TM 10 |  |
| ATACTGACCTTCACCAGTCTGACCTCCATAATGAATGACTTTGCAAACGGATTGGTGTGGAAGATTTCTGGAGGCGTCGTCATCCTGGTG | 1643 |
| *I L T F T S L T S I M N D F A N G L V W K I S G G V V I L V* | 480 |
| GTTTGTGCAATCAACATGTACTTTGTTGTGGTTTACGTCACAGCGCTGAACAGTGTGCTGCTCTACGTTCTTGCTGCTATATTCTCCATT | 1733 |
| *V C A I N M Y F V V V Y V T A L N S V L L Y V L A A I F S I* | 510 |
| TM 11 |  |
| GCCTATCTTTGCTTTGTGGGCTACCTGGTATGGCACTGTTTGGTTGCTTTGGGAGTTTCCTGCCTGGACTTTGGCAGCAGGGTAAGCAAT | 1823 |
| *A Y L C F V G Y L V W H C L V A L G V S C L D F G S R V S N* | 540 |
| TM 12 |  |
| CGACCTGCTGTGTTCATAGAGGAGCCGTATGAGTACGACTCCTAGAGCTGAACAGGACAGTAACCACCAGCATTCCTCTTGGAGACACTG | 1913 |
| *R P A V F I E E P Y E Y D S **  AGCCCTGTACACAGCCAGGACTCTTCTAGATCATATATGATCTTGAGAGCACATTTTCTGCTAATGTCACATGTTTGTCTTGTGTAGGGC  TGGGTCTCAGAGTTTGGTGCTTTTTATGTACCCAGTGAATCAAATTAATACTACACCAGCAATCAGAAAATCAGTTGTCAAATGAATAAT  TGTTACCGTCCTCCTACCGACATACTGAGTAGTTACTATGCTGATGATTAAACCAAGTATCTGTTATGTAAAATAAGATTTTCCATCATT  TTTATAAAATGTAAGCTAGACTTAAAAAACAGACTAGACTAAAACATAGCATCTCAGCTTGTATTTTTATGTATTTATGTCGGTTTATGT  AAACTACAGTAGTAACCACAATGTGTGGGGCTACATTGCTACAACTTCTTTAAAAGAAGCTCAACGTTTTTGTGTTTAATTAGGATTAAA  TAACCTCATAATTACAGAATTGTCACAATTGAAATAAAAACTATTCAAACTATCAAAAAAAAAAAAAAAAAAAA | 554  2003  2093  2183  2273  2363  2437 |

**C**

| ACGTCAGAGAGTCATTGTGACATCTAGTGAC | 3100 |
| --- | --- |
| AAAAAAAAAAGGGATGATAAAGGCTGAGTCATATAAAAGCAGAGTCCTTGTTAGGACACAGTGGACTTTAGGACCAGCAGTGAGTTGGAG  ATGAAAGCCGAGCAAGACGGAGACCTCCTCGAAGAGGACTCTCCTCAGGACAATGGAGTCCAGACAAACCAGTACAGCTCCATCTCTCCT | 121  2110 |
| *M K E G K N E K S T L K D S P Q D N G V Q T N Q Y S S I S P* | 30 |
| CCAGCCTCACCTGTGGCCCAGGAGGAACCTTTTTCTACATACTTTGAAGACAAGGTTGCCATTCCCGAAAATGTCAACCAGGTATTCAGT | 3010 |
| *P A S P V A Q E E P F S T Y F E D K V A I P E N V N Q V F S* | 60 |
| TTCCGAAAACTCTGGGCATTCACTGGACCAGGGTTTTTGATGAGCATTGCTTACTTGGATCCAGGAAACATCGAGTCTGACCTGCAGTCT | 3910 |
| *F R K L W A F T G P G F L M S I A Y L D P G N I E S D L Q S* | 90 |
| TM 1 |  |
| GGAGCTAAAGCAGGCTTTAAGCTCCTGTGGGTGCTACTTGTGGCCACCATCATTGGGCTGCTGTTGCAGAGGTTAGCTGCACGCCTTGGT | 4810 |
| *G A K A G F K L L W V L L V A T I I G L L L Q R L A A R L G* | 120 |
| TM 2 |  |
| GTCGTCACTGGGATGCACCTGGCTGAAGTCTGCAACCGCCAATATCCCACAGTTCCTCGGGTCATCCTTTGGCTGATGGTGGAACTGGCA | 5710 |
| *V V T G M H L A E V C N R Q Y P T V P R V I L W L M V E L A* | 150 |
| ATTATTGGCTCAGACATGCAGGAGGTCATTGGCTGTGCCATTGCTCTCAACCTTCTCTCTGTGGGAAGGATTCCACTGTGGGCAGGAGTC | 6610 |
| *I I G S D M Q E V I G C A I A L N L L S V G R I P L W A G V*  TM 3 | 180 |
| CTCATCACCATCACAGACACATTCGTGTTCCTTTTTCTAGACAAATACGGCCTGAGGAAACTTGAAGCTTTCTTTGGATTTCTCATCACT | 7510 |
| *L I T I T D T F V F L F L D K Y G L R K L E A F F G F L I T* | 210 |
| TM 4 |  |
| GTAATGGCTCTTAGCTTCGGTTACGAGTATGTGCTGGTAAAGCCAGATCAAGGGGAACTTCTGAAGGGGATGTTCTTACCTTACTGCGCC | 8410 |
| *V M A L S F G Y E Y V L V K P D Q G E L L K G M F L P Y C A*  TM 5 | 240 |
| GGCTGTGGGCCTGTGCAGCTGGAGCAGGCAGTGGGCATCGTAGGTGCTGTCATCATGCCCCACAACATCTACTTGCACTCAGCGCTGGTC | 9310 |
| *G C G P V Q L E Q A V G I V G A V I M P H N I Y L H S A L V* | 270 |
| TM 6 |  |
| AAGTCTCGAGACATCGATCGGAAAAATAAGAAGGAAGTAAAGGAAGCCAACAAGTACTACTTCATTGAGTCAACTATTGCTCTCTTTATC | 1021 |
| *K S R D I D R K N K K E V K E A N K Y Y F I E S T I A L F I* | 300 |
| TCCTTCCTCATCAACGTCTTTGTCGTAGCGGTCTTTGCTGAGGCCTTCTACAATAAAACCAATATGGAAGTGCATGAATTTTGCAATAAA | 1111 |
| *S F L I N V F V V A V F A E A F Y N K T N M E V H E F C N K* | 330 |
| TM 7 |  |
| ACTGGCAGCCCTCACTCAGGTCTTTTCCCACAAGACAACAACACACTGGAGGTGGACATCTACAAAGGGGGAGTGGTTCTGGGCTGTGTC | 1201 |
| *T G S P H S G L F P Q D N N T L E V D I Y K G G V V L G C V* | 360 |
| TTTGGCCCTGCAGCCCTCTACATCTGGGCAATAGGGATCCTGGCAGCTGGACAGAGTTCCACCATGACAGGCACTTACTCTGGGCAGTTT | 1291 |
| *F G P A A L Y I W A I G I L A A G Q S S T M T G T Y S G Q F* | 390 |
| TM 8 CTM |  |
| GTTATGGAGGGTTTCCTGAACCTACGGTGGTCCCGTTTTGCTCGGGTGCTGCTGACCCGCTCCATCGCCATCACGCCAACACTGCTGGTA | 1381 |
| *V M E G F L N L R W S R F A R V L L T R S I A I T P T L L V* | 420 |
| TM 9 |  |
| GCCATTTTCCAGGATGTTCAGCATCTGACAGGGATGAACGACTTCCTCAACGTGCTTCAAAGCATGCAGCTTCCGTTTGCTTTGATACCA | 1471 |
| *A I F Q D V Q H L T G M N D F L N V L Q S M Q L P F A L I P* | 450 |
| TM 10 |  |
| ATACTGACCTTCACCAGTCTGACCTCCATAATGAATGACTTTGCAAACGGATTGGTGTGGAAGATTTCTGGAGGCGTCGTCATCCTGGTG | 1561 |
| *I L T F T S L T S I M N D F A N G L V W K I S G G V V I L V* | 480 |
| GTTTGTGCAATCAACATGTACTTTGTTGTGGTTTACGTCACAGCGCTGAACAGTGTGCTGCTCTACGTTCTTGCTGCTATATTCTCCATT | 1651 |
| *V C A I N M Y F V V V Y V T A L N S V L L Y V L A A I F S I* | 510 |
| TM 11 |  |
| GCCTATCTTTGCTTTGTGGGCTACCTGGTATGGCACTGTTTGGTTGCTTTGGGAGTTTCCTGCCTGGACTTTGGCAGCAGGGTAAGCAAT | 1741 |
| *A Y L C F V G Y L V W H C L V A L G V S C L D F G S R V S N* | 540 |
| TM 12 |  |
| CGACCTGCTGTGTTCATAGAGGAGCCGTATGAGTACGACTCCTAGAGCTGAACAGGACAGTAACCACCAGCATTCCTCTTGGAGACACTG | 1831 |
| *R P A V F I E E P Y E Y D S **  AGCCCTGTACACAGCCAGGACTCTTCTAGATCATATATGATCTTGAGAGCACATTTTCTGCTAATGTCACATGTTTGTCTTGTGTAGGGC  TGGGTCTCAGAGTTTGGTGCTTTTTATGTACCCAGTGAATCAAATTAATACTACACCAGCAATCAGAAAATCAGTTGTCAAATGAATAAT  TGTTACCGTCCTCCTACCGACATACTGAGTAGTTACTATGCTGATGATTAAACCAAGTATCTGTTATGTAAAATAAGATTTTCCATCATT  TTTATAAAATGTAAGCTAGACTTAAAAAACAGACTAGACTAAAACATAGCATCTCAGCTTGTATTTTTATGTATTTATGTCGGTTTATGT  AAACTACAGTAGTAACCACAATGTGTGGGGCTACATTGCTACAACTTCTTTAAAAGAAGCTCAACGTTTTTGTGTTTAATTAGGATTAAA  TAACCTCATAATTACAGAATTGTCACAATTGAAATAAAAACTATTCAAACTATCAAAAAAAAAAAAAAAAAAAA | 554  1921  2011  2101  2191  2281  2355 |

**D**

| TTCCTTCTTGTGCTTATTATGAG  AAAGCATGAAGTTTCTATACAAAACTAATCTTTTTAGCTCAATACTTGCATGATAATTACTGTTTTACCCTCAACAGACATGTCTTTCAT | 23  1130 |
| --- | --- |
| GCTCCTTCAGATTATGTAATTGTTGTTTTCTTATTCCAGGGAGGCTGATATTCATCCCTCAAGTTCCCAGGGATACCTCAGCTTGCCCTG  ATGAAAGCCGAGCAAGACGGAGACCTCCTCGAAATGAAGGAAGGAAAGAATGAAAAGTCCACTCTCAAGGACTCTCCTCAGGACAATGGA | 203  293 |
| *M K A E Q D G D L L E E D S P Q D N G V Q T N Q Y S S I S P* | 30 |
| CCAGCCTCACCTGTGGCCCAGGAGGAACCTTTTTCTACATACTTTGAAGACAAGGTTGCCATTCCCGAAAATGTCAACCAGGTATTCAGT | 3830 |
| *P A S P V A Q E E P F S T Y F E D K V A I P E N V N Q V F S* | 60 |
| TTCCGAAAACTCTGGGCATTCACTGGACCAGGGTTTTTGATGAGCATTGCTTACTTGGATCCAGGAAACATCGAGTCTGACCTGCAGTCT | 4730 |
| *F R K L W A F T G P G F L M S I A Y L D P G N I E S D L Q S* | 90 |
| TM 1 |  |
| GGAGCTAAAGCAGGCTTTAAGCTCCTGTGGGTGCTACTTGTGGCCACCATCATTGGGCTGCTGTTGCAGAGGTTAGCTGCACGCCTTGGT | 5630 |
| *G A K A G F K L L W V L L V A T I I G L L L Q R L A A R L G* | 120 |
| TM 2 |  |
| GTCGTCACTGGGATGCACCTGGCTGAAGTCTGCAACCGCCAATATCCCACAGTTCCTCGGGTCATCCTTTGGCTGATGGTGGAACTGGCA | 6530 |
| *V V T G M H L A E V C N R Q Y P T V P R V I L W L M V E L A* | 150 |
| ATTATTGGCTCAGACATGCAGGAGGTCATTGGCTGTGCCATTGCTCTCAACCTTCTCTCTGTGGGAAGGATTCCACTGTGGGCAGGAGTC | 7430 |
| *I I G S D M Q E V I G C A I A L N L L S V G R I P L W A G V*  TM 3 | 180 |
| CTCATCACCATCACAGACACATTCGTGTTCCTTTTTCTAGACAAATACGGCCTGAGGAAACTTGAAGCTTTCTTTGGATTTCTCATCACT | 8330 |
| *L I T I T D T F V F L F L D K Y G L R K L E A F F G F L I T* | 210 |
| TM 4 |  |
| GTAATGGCTCTTAGCTTCGGTTACGAGTATGTGCTGGTAAAGCCAGATCAAGGGGAACTTCTGAAGGGGATGTTCTTACCTTACTGCGCC | 9230 |
| *V M A L S F G Y E Y V L V K P D Q G E L L K G M F L P Y C A*  TM 5 | 240 |
| GGCTGTGGGCCTGTGCAGCTGGAGCAGGCAGTGGGCATCGTAGGTGCTGTCATCATGCCCCACAACATCTACTTGCACTCAGCGCTGGTC | 1013 |
| *G C G P V Q L E Q A V G I V G A V I M P H N I Y L H S A L V* | 270 |
| TM 6 |  |
| AAGTCTCGAGACATCGATCGGAAAAATAAGAAGGAAGTAAAGGAAGCCAACAAGTACTACTTCATTGAGTCAACTATTGCTCTCTTTATC | 1103 |
| *K S R D I D R K N K K E V K E A N K Y Y F I E S T I A L F I* | 300 |
| TCCTTCCTCATCAACGTCTTTGTCGTAGCGGTCTTTGCTGAGGCCTTCTACAATAAAACCAATATGGAAGTGCATGAATTTTGCAATAAA | 1193 |
| *S F L I N V F V V A V F A E A F Y N K T N M E V H E F C N K* | 330 |
| TM 7 |  |
| ACTGGCAGCCCTCACTCAGGTCTTTTCCCACAAGACAACAACACACTGGAGGTGGACATCTACAAAGGGGGAGTGGTTCTGGGCTGTGTC | 1283 |
| *T G S P H S G L F P Q D N N T L E V D I Y K G G V V L G C V* | 360 |
| TTTGGCCCTGCAGCCCTCTACATCTGGGCAATAGGGATCCTGGCAGCTGGACAGAGTTCCACCATGACAGGCACTTACTCTGGGCAGTTT | 1373 |
| *F G P A A L Y I W A I G I L A A G Q S S T M T G T Y S G Q F* | 390 |
| TM 8 CTM |  |
| GTTATGGAGGGTTTCCTGAACCTACGGTGGTCCCGTTTTGCTCGGGTGCTGCTGACCCGCTCCATCGCCATCACGCCAACACTGCTGGTA | 1463 |
| *V M E G F L N L R W S R F A R V L L T R S I A I T P T L L V* | 420 |
| TM 9 |  |
| GCCATTTTCCAGGATGTTCAGCATCTGACAGGGATGAACGACTTCCTCAACGTGCTTCAAAGCATGCAGCTTCCGTTTGCTTTGATACCA | 1553 |
| *A I F Q D V Q H L T G M N D F L N V L Q S M Q L P F A L I P* | 450 |
| TM 10 |  |
| ATACTGACCTTCACCAGTCTGACCTCCATAATGAATGACTTTGCAAACGGATTGGTGTGGAAGATTTCTGGAGGCGTCGTCATCCTGGTG | 1643 |
| *I L T F T S L T S I M N D F A N G L V W K I S G G V V I L V* | 480 |
| GTTTGTGCAATCAACATGTACTTTGTTGTGGTTTACGTCACAGCGCTGAACAGTGTGCTGCTCTACGTTCTTGCTGCTATATTCTCCATT | 1733 |
| *V C A I N M Y F V V V Y V T A L N S V L L Y V L A A I F S I* | 510 |
| TM 11 |  |
| GCCTATCTTTGCTTTGTGGGCTACCTGGTATGGCACTGTTTGGTTGCTTTGGGAGTTTCCTGCCTGGACTTTGGCAGCAGGACACAAATG | 1823 |
| *A Y L C F V G Y L V W H C L V A L G V S C L D F G S R T Q M* | 540 |
| TM 12 |  |
| GGATTCTCTCGGCACACAGACATTTACTTAATGAGTGACATGGACACTGATAATCTGGTAGAGAGATAGGAGGACACAAAGTGAACTGTG | 1913 |
| *G F S R H T D I Y L M S D M D T D N L V E R **  GCGAGGACGACCTGACGACCGGAAGCATGGGACCACTGAATATCGGCCAGTATGTGTTTTTTGTCTTCACCTCTCTGTGCCTGACGTGTT  ATTTTAAAGTGTCCTTACAGCACACAGTGTAGAGCGCCCACAGATGTCCACTCCTGTTCCAAACGGCGATGGATGTTCTTCATCACAAAC  GTCTCAACAACCTCAGCTAAATAATGTTTATGCAAATAATGGTTAAATATCTGTGCTCCAAAGAACTGGACCACTACAAGTGAATAAAAC  AATGACTTCTGTTAAAAAAAAAAAAAAA | 562  2003  2093  2183  2211 |

**E**

| ACGTCAGAGAGTCATTGTGACATCTAGTGAC | 310**0** |
| --- | --- |
| AAAAAAAAAAGGGATGATAAAGGCTGAGTCATATAAAAGCAGAGTCCTTGTTAGGACACAGTGGACTTTAGGACCAGCAGTGAGTTGGAG  ATGAAAGCCGAGCAAGACGGAGACCTCCTCGAAGAGGACTCTCCTCAGGACAATGGAGTCCAGACAAACCAGTACAGCTCCATCTCTCCT | 121  2110 |
| *M K E G K N E K S T L K D S P Q D N G V Q T N Q Y S S I S P* | 30 |
| CCAGCCTCACCTGTGGCCCAGGAGGAACCTTTTTCTACATACTTTGAAGACAAGGTTGCCATTCCCGAAAATGTCAACCAGGTATTCAGT | 3010 |
| *P A S P V A Q E E P F S T Y F E D K V A I P E N V N Q V F S* | 60 |
| TTCCGAAAACTCTGGGCATTCACTGGACCAGGGTTTTTGATGAGCATTGCTTACTTGGATCCAGGAAACATCGAGTCTGACCTGCAGTCT | 3910 |
| *F R K L W A F T G P G F L M S I A Y L D P G N I E S D L Q S* | 90 |
| TM 1 |  |
| GGAGCTAAAGCAGGCTTTAAGCTCCTGTGGGTGCTACTTGTGGCCACCATCATTGGGCTGCTGTTGCAGAGGTTAGCTGCACGCCTTGGT | 4810 |
| *G A K A G F K L L W V L L V A T I I G L L L Q R L A A R L G* | 120 |
| TM 2 |  |
| GTCGTCACTGGGATGCACCTGGCTGAAGTCTGCAACCGCCAATATCCCACAGTTCCTCGGGTCATCCTTTGGCTGATGGTGGAACTGGCA | 5710 |
| *V V T G M H L A E V C N R Q Y P T V P R V I L W L M V E L A* | 150 |
| ATTATTGGCTCAGACATGCAGGAGGTCATTGGCTGTGCCATTGCTCTCAACCTTCTCTCTGTGGGAAGGATTCCACTGTGGGCAGGAGTC | 6610 |
| *I I G S D M Q E V I G C A I A L N L L S V G R I P L W A G V*  TM 3 | 180 |
| CTCATCACCATCACAGACACATTCGTGTTCCTTTTTCTAGACAAATACGGCCTGAGGAAACTTGAAGCTTTCTTTGGATTTCTCATCACT | 7510 |
| *L I T I T D T F V F L F L D K Y G L R K L E A F F G F L I T* | 210 |
| TM 4 |  |
| GTAATGGCTCTTAGCTTCGGTTACGAGTATGTGCTGGTAAAGCCAGATCAAGGGGAACTTCTGAAGGGGATGTTCTTACCTTACTGCGCC | 8410 |
| *V M A L S F G Y E Y V L V K P D Q G E L L K G M F L P Y C A*  TM 5 | 240 |
| GGCTGTGGGCCTGTGCAGCTGGAGCAGGCAGTGGGCATCGTAGGTGCTGTCATCATGCCCCACAACATCTACTTGCACTCAGCGCTGGTC | 9310 |
| *G C G P V Q L E Q A V G I V G A V I M P H N I Y L H S A L V* | 270 |
| TM 6 |  |
| AAGTCTCGAGACATCGATCGGAAAAATAAGAAGGAAGTAAAGGAAGCCAACAAGTACTACTTCATTGAGTCAACTATTGCTCTCTTTATC | 1021 |
| *K S R D I D R K N K K E V K E A N K Y Y F I E S T I A L F I* | 300 |
| TCCTTCCTCATCAACGTCTTTGTCGTAGCGGTCTTTGCTGAGGCCTTCTACAATAAAACCAATATGGAAGTGCATGAATTTTGCAATAAA | 1111 |
| *S F L I N V F V V A V F A E A F Y N K T N M E V H E F C N K* | 330 |
| TM 7 |  |
| ACTGGCAGCCCTCACTCAGGTCTTTTCCCACAAGACAACAACACACTGGAGGTGGACATCTACAAAGGGGGAGTGGTTCTGGGCTGTGTC | 1201 |
| *T G S P H S G L F P Q D N N T L E V D I Y K G G V V L G C V* | 360 |
| TTTGGCCCTGCAGCCCTCTACATCTGGGCAATAGGGATCCTGGCAGCTGGACAGAGTTCCACCATGACAGGCACTTACTCTGGGCAGTTT | 1291 |
| *F G P A A L Y I W A I G I L A A G Q S S T M T G T Y S G Q F* | 390 |
| TM 8 CTM |  |
| GTTATGGAGGGTTTCCTGAACCTACGGTGGTCCCGTTTTGCTCGGGTGCTGCTGACCCGCTCCATCGCCATCACGCCAACACTGCTGGTA | 1381 |
| *V M E G F L N L R W S R F A R V L L T R S I A I T P T L L V* | 420 |
| TM 9 |  |
| GCCATTTTCCAGGATGTTCAGCATCTGACAGGGATGAACGACTTCCTCAACGTGCTTCAAAGCATGCAGCTTCCGTTTGCTTTGATACCA | 1471 |
| *A I F Q D V Q H L T G M N D F L N V L Q S M Q L P F A L I P* | 450 |
| TM 10 |  |
| ATACTGACCTTCACCAGTCTGACCTCCATAATGAATGACTTTGCAAACGGATTGGTGTGGAAGATTTCTGGAGGCGTCGTCATCCTGGTG | 1561 |
| *I L T F T S L T S I M N D F A N G L V W K I S G G V V I L V* | 480 |
| GTTTGTGCAATCAACATGTACTTTGTTGTGGTTTACGTCACAGCGCTGAACAGTGTGCTGCTCTACGTTCTTGCTGCTATATTCTCCATT | 1651 |
| *V C A I N M Y F V V V Y V T A L N S V L L Y V L A A I F S I* | 510 |
| TM 11 |  |
| GCCTATCTTTGCTTTGTGGGCTACCTGGTATGGCACTGTTTGGTTGCTTTGGGAGTTTCCTGCCTGGACTTTGGCAGCAGGACACAAATG | 1741 |
| *A Y L C F V G Y L V W H C L V A L G V S C L D F G S R V S N* | 540 |
| TM 12 |  |
| GGATTCTCTCGGCACACAGACATTTACTTAATGAGTGACATGGACACTGATAATCTGGTAGAGAGATAGGAGGACACAAAGTGAACTGTG | 1831 |
| *G F S R H T D I Y L M S D M D T D N L V E R **  GCGAGGACGACCTGACGACCGGAAGCATGGGACCACTGAATATCGGCCAGTATGTGTTTTTTGTCTTCACCTCTCTGTGCCTGACGTGTT  ATTTTAAAGTGTCCTTACAGCACACAGTGTAGAGCGCCCACAGATGTCCACTCCTGTTCCAAACGGCGATGGATGTTCTTCATCACAAAC  GTCTCAACAACCTCAGCTAAATAATGTTTATGCAAATAATGGTTAAATATCTGTGCTCCAAAGAACTGGACCACTACAAGTGAATAAAAC  AATGACTTCTGTTAAAAAAAAAAAAAAA | 562  1921  2011  2101  2129 |
