# Supplementary material for: Natural history of SLC11 genes in vertebrates: tales from the fish world
Source: BMC Evol Biol. 2011 Apr 18;11:106. doi: 10.1186/1471-2148-11-106 (PMC3103463; doi:10.1186/1471-2148-11-106)
Supplement: Additional file 2 — Figure S2: Comparative view of the genomic structure, organization and size of SLC11 homologs of several species. This file contains a comparative view of the genomic structure and size of sea bass slc11a2-α and slc11a2-β with homologs from other fishes, amphibians, mammals and insects. [file 1471-2148-11-106-S2.DOC]

**Additional File 2, Figure S2 – Comparative view of the genomic structure, organization and size of *SLC11* homologs of several species.** Exons are represented as black boxes, introns as lines and untranslated regions (UTR) as white boxes.
