# Supplementary material for: Natural history of SLC11 genes in vertebrates: tales from the fish world
Source: BMC Evol Biol. 2011 Apr 18;11:106. doi: 10.1186/1471-2148-11-106 (PMC3103463; doi:10.1186/1471-2148-11-106)

**Additional File 3, Figure S3 – Phylogenetic trees.** Based on the deduced amino acid sequences of SLC11A1 and SLC11A2 homologs of several species. The trees were constructed using (A) Bayesian inference with MrBayes, with a model of gamma-distributed rate variation across sites and a proportion of invariable sites, (B) neighbor-joining method, with the Poisson model and pairwise deletion of gaps and (C) maximum-parsimony method, with complete deletion of gaps and Close-Neighbor-Interchange (CNI) on Random Trees search method, with MEGA5. Numbers on nodes indicate (A) branch lengths or (B,C) frequency with which the node was recovered per 100 bootstrap replications in a total of 1000.


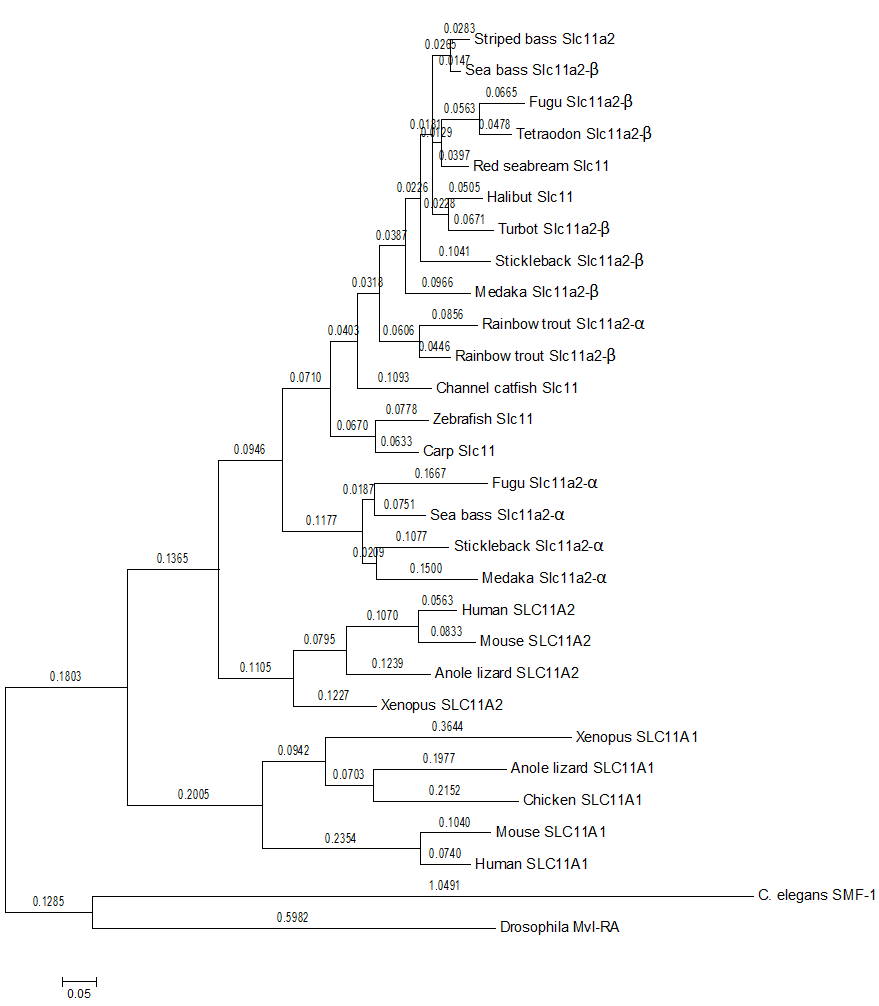
**A**

**B**


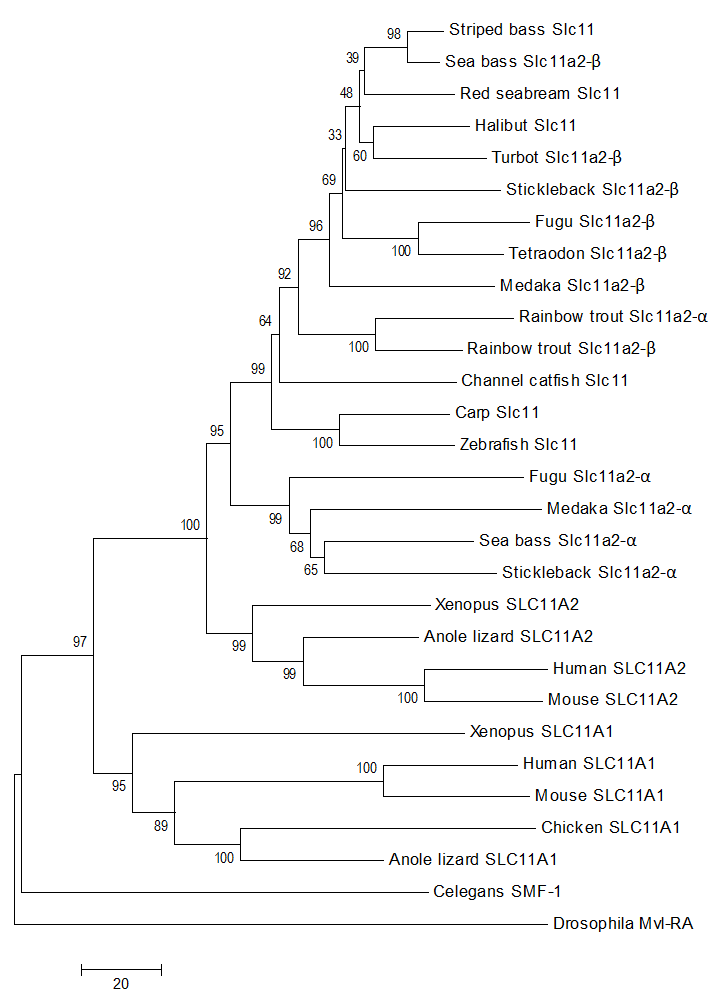


**C**


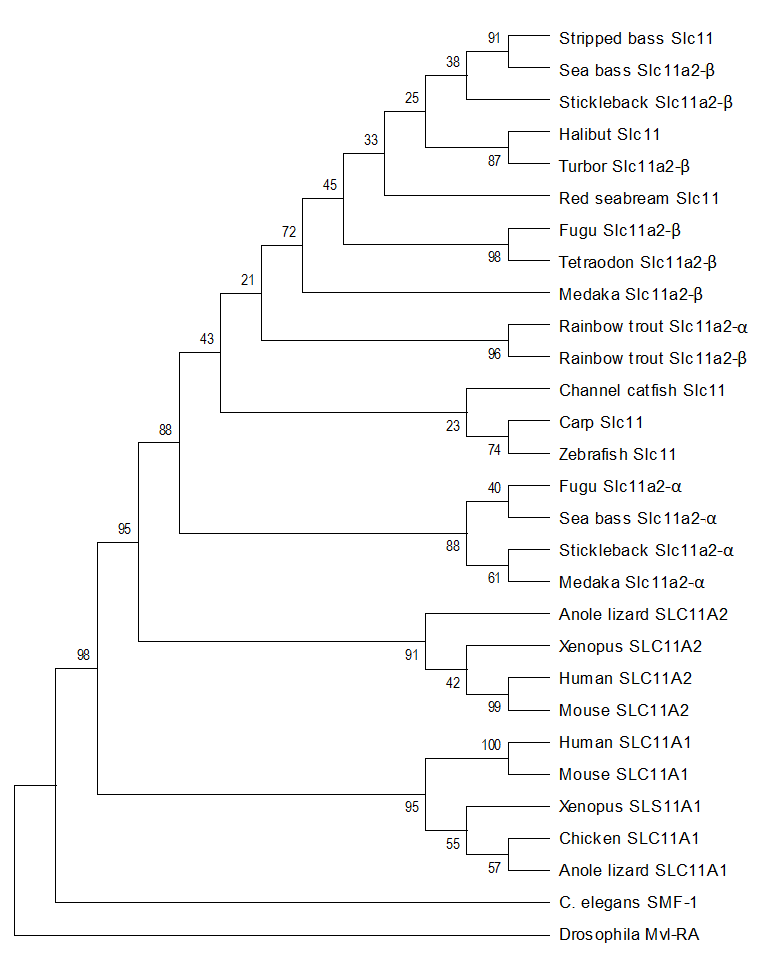

Supplement: Additional file 3 — Figure S3: Additional phylogenetic trees. This file contains additional phylogenetic trees constructed with Bayesian, neighbour-joining and maximum-parsimony methods. [file 1471-2148-11-106-S3.DOC]
