# Supplementary material for: Natural history of SLC11 genes in vertebrates: tales from the fish world
Source: BMC Evol Biol. 2011 Apr 18;11:106. doi: 10.1186/1471-2148-11-106 (PMC3103463; doi:10.1186/1471-2148-11-106)
Supplement: Additional file 4 — Figure S4: Evolutionary relationships of AGAP2, ANKRD52 and MARCH9 gene families. This file contains phylogenetic trees for AGAP2, ANKRD52 and MARCH9, constructed with the maximum-likelihood method. [file 1471-2148-11-106-S4.DOC]

**Additional File 4, Figure S4 - Evolutionary relationships of (A) AGAP2, (B) ANKRD52 and (C) MARCH9 gene families.** The evolutionary history was inferred by using the Maximum Likelihood method based on the JTT matrix-based model. The bootstrap consensus tree inferred from 1000 replicates is taken to represent the evolutionary history of the taxa analyzed. Initial tree(s) for the heuristic search were obtained automatically as follows. When the number of common sites was < 100 or less than one fourth of the total number of sites, the maximum parsimony method was used; otherwise BIONJ method with MCL distance matrix was used. The trees are drawn to scale, with branch lengths measured in the number of substitutions per site (above the branches). The analysis involved 12 (AGAP2), 24 (ANKRD52) and 25 (MARCH9) amino acid sequences. All positions containing gaps and missing data were eliminated. There were a total of 665 (AGAP2), 471 (ANKRD52) and 180 (MARCH9) positions in the final dataset. Arrow indicates the duplication point of α and β isoforms in teleosts (except in MARCH9).

**A**

**B**

**C**
