# Supplementary material for: Natural history of SLC11 genes in vertebrates: tales from the fish world
Source: BMC Evol Biol. 2011 Apr 18;11:106. doi: 10.1186/1471-2148-11-106 (PMC3103463; doi:10.1186/1471-2148-11-106)

**Additional File 5, Figure S5 - Sea bass mortality during experimental infection, at 12 hours intervals.** Survival of fish i.p. injected with TSB (□) or 1.0×10^5^ *Photobacterium damselae*/fish (■). Arrows indicate time points for collection of samples.


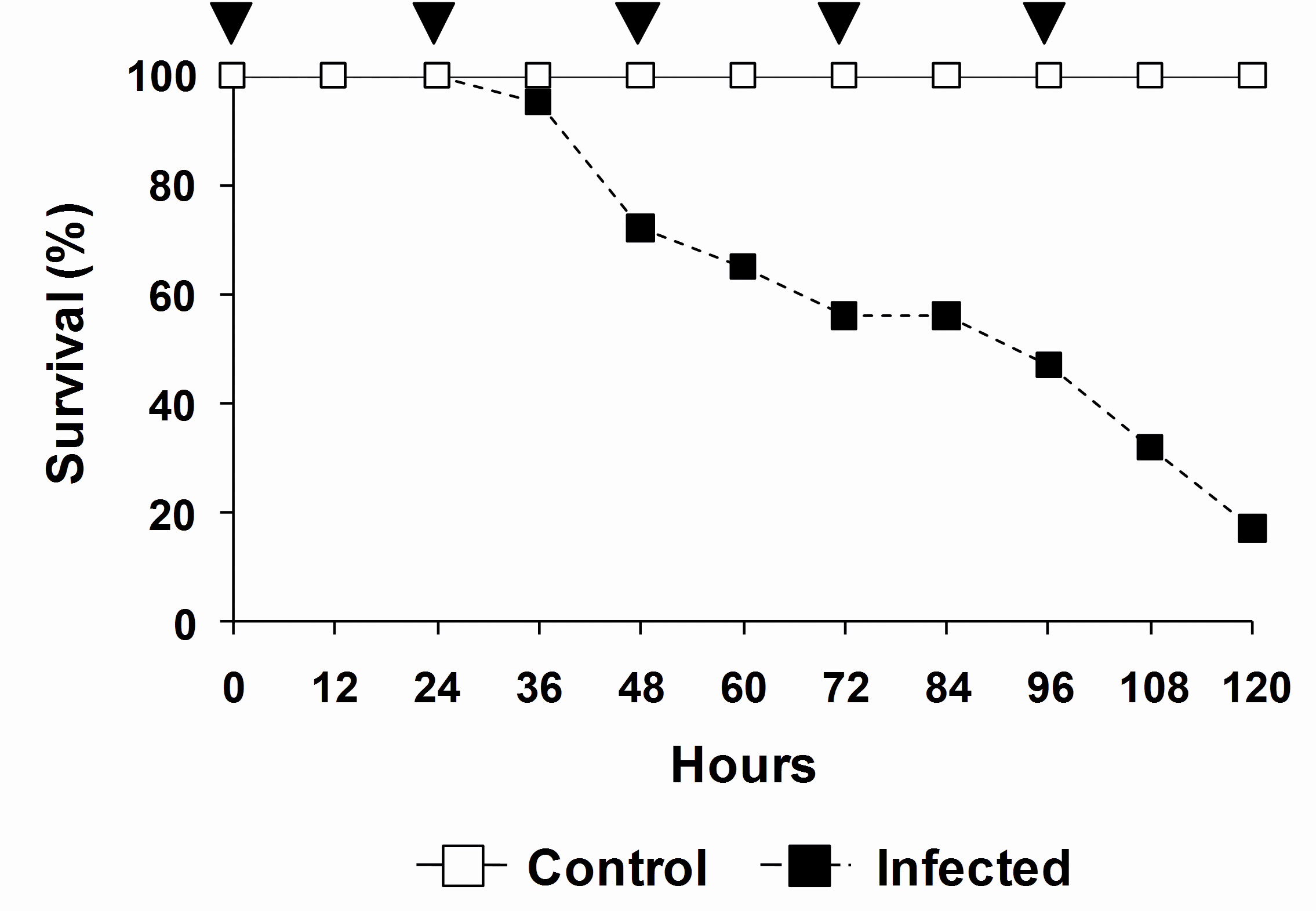

Supplement: Additional file 5 — Figure S5: Sea bass mortality during experimental infection. This file contains a graphic showing sea bass mortality during infection with Photobacterium damselae, at 12 hour intervals. [file 1471-2148-11-106-S5.DOC]
