# Supplementary material for: Natural history of SLC11 genes in vertebrates: tales from the fish world
Source: BMC Evol Biol. 2011 Apr 18;11:106. doi: 10.1186/1471-2148-11-106 (PMC3103463; doi:10.1186/1471-2148-11-106)
Supplement: Additional file 6 — Table S1: Primers used in this study. This file contains a table with all the primers used for sequencing, southern blot, in situ hybridization and gene expression analysis performed in this study. [file 1471-2148-11-106-S6.DOCX]

**Additional File 6, Table S1- List of primers used for sequencing, PCR and probes.**

| **Primer name** | **Primer sequence (5' 🡪 3')** | **Primer use** |
| --- | --- | --- |
| Alpha-For | CGCGTTCAACCTCCTCTCCTCT | Real-time PCR |
| Alpha-Rev | AGCCCTCGCAGTACGGCACA | Real-time PCR |
| Beta-For | TGCTCTCAACCTTCTCTCTGTG | Real-time PCR |
| Beta-Rev | AGCCGGCGCAGTAAGGTAAG | Real-time PCR |
| BetaExon1A-For | AAGACGGAGACCTCCTCGAA | RT-PCR |
| BetaExon1B-For | GGAAGGAAAGAATGAAAAGTCCAC | RT-PCR |
| BetaExon15-Rev | TGAACACAGCAGGTCGATTGC | RT-PCR |
| BetaExon16-Rev | TGCCGAGAGAATCCCATTTGTGT | RT-PCR |
| SouthernProbe-For | GTGCTGGTAAAGCCAGAT | Southern probe |
| SouthernProbe-Rev | CACTCAGCGCTGGTCAA | Southern probe |
| AlphaProbeISH-For | CGACCATCGCGCTGTTCG | *In situ* hybridization |
| AlphaProbeISH-Rev | CACCTCTAGAGTTTCATTGTTCA | *In situ* hybridization |
| BetaProbeISH-For | TCAACTATTGCTCTCTTTATCTCC | *In situ* hybridization |
| BetaProbeISH-Rev | ACCTCCAGTGTGTTGTTGTCTT | *In situ* hybridization |
| Nramp-For | AAGACATGCAGGAGGTCAT | Sequencing |
| Nramp-Rev | ATGTTGTGGGGCATGATGA | Sequencing |
| Alpha2-For | CCTCATTCCCATCCTCACG | Sequencing |
| Alpha2-Rev | CGTGAGGATGGGAATGAGG | Sequencing |
| Alpha3-For | ATCTCTGTTCCCTCTGAACAATG | Sequencing |
| Alpha3-Rev | CACCTCTAGAGTTTCATTGTTCA | Sequencing |
| Alpha4-For | GCTCTACATCTGGGCCGTG | Sequencing |
| Alpha4-Rev | AGCTGCAAGGATCCCCACG | Sequencing |
| Alpha5-For | TTTGTGCCGTACTGCGAG | Sequencing |
| Beta1-For | TCAGGACAATGGAGTCCAGA | Sequencing |
| Beta1-Rev | CAATGACCTCCTGCATGTCT | Sequencing |
| Beta2-For | TGGGCATCGTAGGTGCTG | Sequencing |
| Beta2-Rev | CGTTCATTATGGATGTCAGACT | Sequencing |
| Beta3-For | GGCTGATATTCATCCCTC | Sequencing |
| Beta3-Rev | AGAGAGAAGGTTGAGAGCAA | Sequencing |
| Beta4-For | GCATGCAGCTTCCGTTTG | Sequencing |
| Beta4-Rev | AGGGCTCAGTGTCTCCAA | Sequencing |
| Beta5-For | TACTTGTGGCCACCATCAT | Sequencing |
| Beta5-Rev | AGCTAAGAGCCATTACAGTG | Sequencing |
| Beta6-For | AACTGGCAGCCCTCACT | Sequencing |
| Beta6-Rev | TTGTAGATGTCCACCTCCA | Sequencing |
| Beta7-For | ACTTACTCTGGGCAGTTTG | Sequencing |
| Beta7-Rev | CAGCAGTGTTGGCGTGA | Sequencing |
| Beta8-For | CTGCTATATTCTCCATTGCC | Sequencing |
| Beta8-Rev | CGACGCCTCCAGAAATC | Sequencing |
| Race5_Univ1-Rev | TGACCTCCTGCATGTCTGAGC | 5' RACE |
| Race5_Univ2-Rev | TTGCAGACTTCAGCCAGGTGC | 5' RACE |
| Race3_Alpha-For | GATGTTTGTGCCGTACTGCGAG | 3' RACE |
| Race3_Univ-For | GATGCACCTGGCTGAAGTCTG | 3' RACE |
| Actin-For | CAGAAGGACAGCTACGT | Real-time / RT-PCR |
| Actin-Rev | GTCATCTTCTCCCTGTTGGC | Real-time / RT-PCR |
